# Structural and functional studies of D109A human αB-crystallin contributing to the development of cataract and cardiomyopathy diseases

**DOI:** 10.1371/journal.pone.0260306

**Published:** 2021-11-29

**Authors:** Mahtab Hafizi, Natalia A. Chebotareva, Maryam Ghahramani, Faezeh Moosavi-Movahedi, Seyed Hossein Khaleghinejad, Boris I. Kurganov, Ali Akbar Moosavi-Movahedi, Reza Yousefi

**Affiliations:** 1 Protein Chemistry Laboratory (PCL), Department of Biology, College of Sciences, Shiraz University, Shiraz, Iran; 2 Bach Institute of Biochemistry, Research Center of Biotechnology of the Russian Academy of Sciences, Moscow, Russia; 3 Institute of Biochemistry and Biophysics (IBB), The University of Tehran, Tehran, Iran; INRA Centre de Jouy-en-Josas, FRANCE

## Abstract

αB-crystallin (heat shock protein β5/HSPB5) is a member of the family of small heat shock proteins that is expressed in various organs of the human body including eye lenses and muscles. Therefore, mutations in the gene of this protein (*CRYAB*) might have many pathological consequences. A new mutation has recently been discovered in the α-crystallin domain of this chaperone protein which replaces aspartate 109 with alanine (D109A). This mutation can cause myofibrillar myopathy (MFM), cataracts, and cardiomyopathy. In the current study, several spectroscopic and microscopic analyses, as well as gel electrophoresis assessment were applied to elucidate the pathogenic contribution of human αB-crystallin bearing D109A mutation in development of eye lens cataract and myopathies. The protein oligomerization, chaperone-like activity and chemical/thermal stabilities of the mutant and wild-type protein were also investigated in the comparative assessments. Our results suggested that the D109A mutation has a significant impact on the important features of human αB-crystallin, including its structure, size of the protein oligomers, tendency to form amyloid fibrils, stability, and chaperone-like activity. Given the importance of aspartate 109 in maintaining the proper structure of the α-crystallin domain, its role in the dimerization and chaperone-like activity, as well as preserving protein stability through the formation of salt bridges; mutation at this important site might have critical consequences and can explain the genesis of myopathy and cataract disorders. Also, the formation of large light-scattering aggregates and disruption of the chaperone-like activity by D109A mutation might be considered as important contributing factors in development of the eye lens opacity.

## Introduction

The heat shock proteins (Hsps) are produced by cells in response to a variety of stressful conditions such as heat, ultraviolet (UV) radiation, cold, and wound, and many members of this family have chaperone-like activity. A distinguished class of Hsp family, small heat shock proteins (sHsp), is consisting of proteins with molecular weights up to 43 kDa [1, 2]. The common feature of all sHsps is the existence of a conserved sequence of 80–100 amino acid residues, forming the α-crystallin domain. Despite this central domain, the marginal N-terminal and C-terminal domains are less conserved in sequence and more variable in structure [3]. The function of sHsps is to interact with unfolded or misfolded proteins in order to prevent aggregate formation and keep them refoldable [1].

α-crystallin is an important member of sHsps expressed in some of the vertebrate’s tissues and has structural roles, as well as chaperone-like activity [4, 5]. This protein exists in the multimeric forms consisting of two homologous proteins, αA and αB-crystallins, having an approximate homology of 57% [5–7]. *CRYAA*, the responsible gene for encoding the 173 amino acid residues of human αA-crystallin, exists on chromosome 21 while its counterpart protein, αB-crystallin, with 175 amino acids is coded by *CRYAB* gene on chromosome 11 [6]. αA-crystallin, known as HSPB4, is almost exclusively expressed in the eye lens where its interaction with αB-crystallin in a ratio of 3:1 allocates 40% of the whole protein combination to itself [8–10]. However, HSPB5 (αB-crystallin) can be found in a wide range of the body organs and tissues such as heart, brain, muscle, kidney and liver [8, 11, 12]. This wide expression of αB-crystallin explains why mutations in this protein can cause disorders in different parts of human body such as muscle or heart, while mutations in αA-crystallin are mostly limited to the cataract development in eye lenses [13, 14].

Mutations in α-crystallin proteins were under investigation during the past decades, and the outcome of these studies is the discovery of the several pathogenic ones, especially missense mutations. Missense mutations in human αB-crystallin can occur in all three regions of this protein, for instance, R11H, P20S and R56W are three cataractogenic mutations in the N-terminal region. Also, mutations as D109H and R120G locating in the α-crystallin domain cause both myopathy and cataract [15–19], while those such as G154S and R157H occurring in the C-terminal extension lead to the myopathy. Aspartate 109 is an important residue in the formation of αB-crystallin dimers as it forms a salt bridge with the arginine 120 in the adjacent monomer. Any missense mutation which results in the elimination of the negative charge of aspartate 109 will disrupt αB-crystallin dimer structure and all its features relying on it [14, 20].

**Fig 1:**
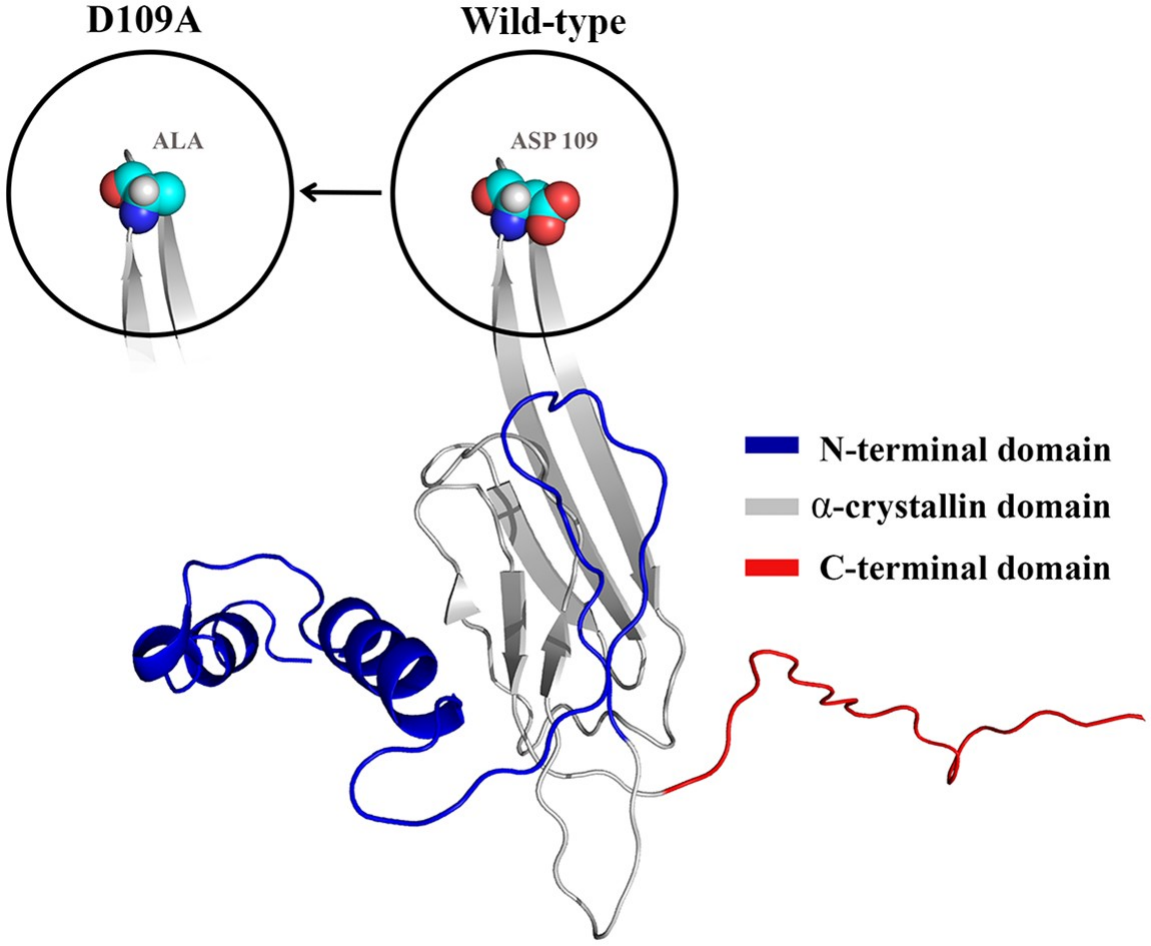
The domain organization of human αB-crystallin and position of D109A mutation in structure of this protein (PDB ID: 3J07).

A recently reported mutation in human αB-crystallin has been identified in patients with myofibrillar myopathy involving aspartate 109 (D109A mutation). The three-domain structural organization and position of this mutation in the protein structure are shown as Fig 1.

In this mutation, the negatively charged aspartate residue is substituted with a neutral alanine and this replacement is expected to have important impacts on the chaperone structure and its anti-aggregation function [21]. In this research, both D109A mutant αB-crystallin and its wild-type counterpart were over-expressed, purified and studied in several experiments using different spectroscopic methods, as well as other techniques in order to gain further complementary information on their structural and functional differences.

## Materials and methods

1-Anilino-8-naphthalene sulfonic acid (ANS), thioflavin T (ThT), bovine pancreatic insulin, bovine liver catalase, chicken egg white lysozyme, α-glucosidase (α-Gls), dithiothreitol (DTT), isopropyl β-D-1-thiogalactopyranoside (IPTG) and kanamycin were purchased from Sigma. The TEM grid was made of copper and provided by Agar Scientific. β-mercaptoethanol (β-ME), ethylenediaminetetraacetic acid (EDTA) and other chemicals were provided by the Merck Company.

### Mutagenesis, expression and purification

The mutagenesis procedure was done using the QuikChange II XL Site-Directed Mutagenesis Kit (Stratagene) in order to substitute adenine 326 with cytosine (326 A>C) in the cDNA of wild-type αB-crystallin which has been cloned in pET-28b(+) to construct the D109A mutation [21, 22]. The validity of the created mutation was confirmed by sequencing. The plasmid containing the wild-type and D109A αB-crystallin cDNA were transformed into the BL21 (DE3) *Escherichia coli* cells. The expression process was done according to the previously applied approach [22]. After protein expression the obtained cell pellets were lysed with three-fold volumes of 40 mM Tris buffer (containing 8 M urea and 10 mM β-ME at pH 8 and 8.2 for the wild-type and mutant proteins, respectively). Then, the final lysate underwent a centrifugation at 10,000 × g for 45 minutes. Also, both supernatant and pellet of these proteins were assessed by SDS-PAGE analysis in order to evaluate their solubility. The purification of the wild-type and mutant proteins were done using a DEAE-cellulose anion exchange column (1.5 cm × 12 cm) with a flow rate of 1 mL.min^-1^. The column was equilibrated with 20 mM Tris buffer (containing 4 M urea and 5 mM β-ME). The used pH of buffer for the wild-type and mutant proteins was 8 and 8.2, respectively. The eluted flow through fractions with the highest protein concentration were pooled [23]. This procedure was repeated two-three times. Finally, the purified proteins which were confirmed by SDS-PAGE analyses (gel 12%) dialyzed against double distilled water. The extinction coefficients (ε_280 nm_) of 0.693 for 1 mg.mL^-1^ human αB-crystallin and D109A mutant protein were used to estimate their concentrations.

### Fluorescence spectroscopic assessments

All samples for fluorescence studies were prepared in 50 mM sodium phosphate buffer, pH 7.4, mentioned as buffer A, with a concentration of 0.15 mg.mL^-1^. Fluorescence spectra were collected at three different temperatures. Also, Trp fluorescence experiment was performed continuously in the temperature range of 25–80 °C using a Varian Cary Eclipse fluorescence spectrophotometer (Australia). For recording Tyr and Trp emission spectra, the excitation wavelength was set at 280 nm and 295 nm, respectively, while the emission spectrum was collected between 300–500 nm. The excitation/emission band passes for Tyr and Trp fluorescence were set at 5/10 nm [22]. The synchronous fluorescence spectra were recorded between 200–350 nm with excitation wavelength steps of 15 nm and 60 nm for Tyr and Trp, respectively [23].

The surface hydrophobicity of the wild-type and D109A αB-crystallins was studied using ANS probe. After a 30 minutes incubation of protein samples (0.15 mg.mL^-1^) with 100 μM ANS in the dark, the ANS fluorescence spectra were collected between 400–600 nm with an excitation wavelength of 365 nm and an excitation and emission bandpass of 10 nm [24]. The concentration of ANS solution was determined at λ = 350 nm using an extinction coefficient of 4950 M^-1^ cm^-1^.

To assess the fibrillogenic features of the proteins, the protein samples (2 mg.mL^-1^) were incubated under thermochemical stress (1 M guanidine hydrochloride (GdnHCl) at 60 °C for 4 days) [25]. At the end of incubation, a 20 μM ThT was added to the protein samples and incubated for 5 minutes in the dark. After that, the protein samples were excited at 440 nm, with an excitation and emission slit of 10 nm. The emission spectra were recorded between 450–600 nm. Also, the concentration of ThT solution was determined by measuring the absorbance at 420 nm, considering a molar extinction coefficient of 24420 M^–1^ cm^–1^.

### Circular dichroism (CD) spectroscopy

The far and near UV-CD spectra of both wild-type and mutant protein samples, prepared in buffer A, were collected at 25 °C using a JASCO J-810 spectropolarimeter instrument as described earlier [22]. The concentration of protein samples was fixed at 0.2 mg.mL^-1^ and 1.5 mg.mL^-1^ for the far and near UV-CD assessments, respectively. The path length for far UV-CD was 0.1 cm, while it was 1 cm for the near UV-CD study. The data were analyzed using the DICHROWEB server with the CONTIN algorithm and were reported as molar ellipticity [26].

### Near-infrared (NIR), Fourier-transform infrared (FTIR) and Raman spectroscopic studies

The NIR and FTIR spectroscopies were also used for further structural analyses. For both studies, 5 mg of homogeneous protein powder was used. The NIR spectra were collected using a NIRS XDS series Vis-NIR spectrometer (Metrohm, Switzerland) with the reflectance mode, in the range of 8,000 to 4,000 cm^-1^ and a resolution of 8 cm^-1^ at room temperature [27]. The FTIR spectra of wild-type and mutant protein were recorded between 1700 to 1600 cm^-1^ with a resolution of 4 cm^-1^ and the accumulation of 256 scans using a Tensor II, Bruker-Germany which was equipped with a diamond attenuated total reflectance (ATR) [23, 28]. Also, the Raman spectra were measured using Raman spectrometer Lab Ram HR (Horiba, Japan) equipped with a confocal microscope using a 532 nm red laser excitation as described previously [23]. The spectra were recorded with acquisition time of 240 s, at 17 mW power laser and optical microscope objective 50x. The estimation of the secondary structural content in amide I region (1700–1600 cm^-1^) were carried out for both FTIR and Raman spectra. The Gaussian function was used for deconvolution analysis which was done using the five peak positions: 1600–1615 cm^-1^ (side chain), 1615–1635 cm^-1^ (β-sheet), 1635–1650 cm^-1^ (disordered), 1650–1665 cm^-1^ (α-helix), and 1665–1700 cm^-1^ (turn) [29, 30].

### Dynamic light scattering

To investigate the effect of temperature on the size of wild-type and mutant protein oligomers, protein samples were prepared in buffer A with a concentration of 1 mg. mL^-1^. The experiment was carried out using a nanoparticle analyzer SZ-100 (Horiba, Japan) with a laser wavelength set at 532 nm and a scattering angle of 173° [31].

### Analytical ultracentrifugation

The samples were dialyzed before the run during 15 hours against 40 mM sodium phosphate buffer, 100 mM NaCl, 1 mM EDTA, 3 mM NaN_3_, pH 7.0, at 4 °C. Sedimentation velocity experiments were carried out at 20 °C in a Model E analytical ultracentrifuge (Beckman), equipped with absorbance optics, a photoelectric scanner, a monochromator and a computer on-line. A four-hole rotor An-F Ti and 12 mm double sector cells were used. The rotor speed was 40,000 rpm. Sedimentation profiles of the proteins were recorded by measuring the absorbance at 280 nm and all cells were scanned simultaneously. The time interval between scans was 2.5 minutes. The concentration of human αB-crystallin and the D109A mutant protein was fixed at 1.0 mg.mL^-1^ and 0.5 mg.mL^-1^, respectively. The differential sedimentation coefficient distributions [ls-g*(*s*) versus *s* and *c*(*s*) versus *s*] were determined using the SEDFIT program [32]. The *c*(*s*) analysis was performed with regularisation at the confidence level of 0.68 and a floating frictional ratio. The ls-g*(*s*) analysis was performed with regularization at the confidence level of 0.9. Weight-average sedimentation coefficients were obtained by integration of the ls-g*(*s*) distribution. The first experiment was performed after dialysis at 20 °C and the second experiment was done after the samples were incubated at 20 °C for 24 hours [32].

### Chemical and thermal denaturation

The chemical stability of the mutant and wild-type human αB-crystallins was investigated to measure the protein structural stability according to the previous study [22]. The protein samples (0.15 mg. mL^-1^) were incubated with an increasing concentrations of urea (0–8 M) for 18 hours and the emission fluorescence of tryptophan residues was recorded. In order to quantify the stability parameters, all profiles were analysed with the aid of a global three state fitting procedure, according to the following equation:

$$F=\frac{F_{\text{N}}+F_{\text{I}}\text{exp}\left\{(-\Delta G_{1}^{0}+m_{1}[\text{urea}])/RT\right\}+F_{\text{U}}\text{exp}\left\{(-\Delta G_{2}^{0}+m_{2}[\text{urea}])/RT\right\}}{1+\text{exp}\left\{(-\Delta G_{1}^{0}+m_{1}[\text{urea}])/RT\right\}+\text{exp}\left\{(-\Delta G_{2}^{0}+m_{2}[\text{urea}])/RT\right\}}$$ (1)

Moreover, the differential scanning calorimetry (DSC) analyses were carried out by a Nano-Differential Scanning Calorimeter II (N-DSC II, Model 6100) with a heating rate of 2 °C/min and 2 atm pressure. The concentration of αB-crystallin and the D109A mutant protein was adjusted at 1.0 mg. mL^-1^. The analysis was done by CpCalc analysis software (CpCalc 2.1) for data assessment [33]. Also, the following equations were used to calculate the thermodynamic parameters [34].


$$\Delta H(T)=\int_{T_{0}}^{T}\Delta C_{p}dT$$ (2)

$$\Delta S(T)=\int_{T_{0}}^{T}\frac{\Delta C_{p}}{T}dT$$ (3)

ΔG(T)=ΔH(T)−TΔS(T) (4)

$$\Delta G(T)=\Delta H(\frac{T_{m}-T}{T_{m}})-\Delta C_{p}\left[T_{m}-T+T\;\text{ln}(\frac{T}{T_{m}})\right]$$ (5)

### Proteolytic study

To study the stability of different αB-crystallin samples against the proteolytic activity of α-chymotrypsin, 1 mg.mL^-1^ of each protein sample, prepared in buffer A, was incubated with 0.01 mg.mL^-1^ of α-chymotrypsin (the enzyme and its substrate must be used in a ratio of 1 to 100 w/w, respectively) for 0, 5, 10 and 15 minutes at 37 °C. Then, 15 μg of each protein sample was loaded into an SDS-PAGE wells [22].

### Fluorescence microscopy

Fluorescence microscopy was used to investigate the fiber plaque of the protein samples (2 mg.mL^-1^) in buffer A before and after incubation at 60 °C in the presence of 1 M GdnHCl for 4 days. After incubation of the protein samples (0.15 mg.mL^-1^) with 20 μM ThT for 5 minutes, they were studied with a Lionheart FX fluorescence microscope (Biotek-USA). The green filter (GPF) was used with an excitation and emission wavelength of 469 nm and 525 nm, respectively [35].

### Transmission electron microscopy (TEM)

The microscopic studies were performed to assess the amyloidogenic propensity of the mutant protein under thermochemical stress. To study the morphology of amyloid fibers, 2 mg.mL^-1^ of protein samples in the presence of 1 M GdnHCl were incubated at 60 °C for 4 days. Then, 15 μL of each sample with a concentration of 1 mg.mL^-1^ was fixed on electron microscopy grids (made of copper) followed by washing with water and 1% uranyl acetate staining.

Imaging was then carried out at 100 kV excitation voltages, using a Philips 906E transmission electron microscope, and the micrographs were analyzed by a MegaView G2 Soft imaging system [25].

### Chaperone-like activity assay

The chaperone like activity of D109A mutant and wild-type αB-crystallins, prepared in buffer A, was studied in two different concentrations (0.1 mg.mL^-1^ and 0.2 mg.mL^-1^), using different client proteins. Bovine pancreatic insulin (0.3 mg.mL^-1^) and chicken egg white lysozyme (0.2 mg.mL^-1^) were used as client proteins which their aggregation was induced using 20 mM DTT at 40 °C. The aggregation of bovine liver catalase (0.3 mg.mL^-1^) was induced at 60 °C [22]. This assay was carried out by recording the light scattering spectra at 400 nm as a function of time (20 minutes, 30 minutes and 60 minutes for bovine pancreatic insulin, chicken egg white lysozyme and bovine liver catalase, respectively) using a T90^+^ UV-Vis spectrophotometer. The chaperone-like activity of protein samples was quantified using Eq 6 in which A_rt_ and A_r0_ represents the area under curve of the aggregation of target proteins in the presence and absence of chaperone, respectively.


$$\%Protection=\left(1-A_{\text{rt}}/A_{\text{r}0}\right)\times 100$$ (6)

### Enzyme thermal inactivation analysis and refolding assessment

In order to study the ability of different αB-crystallin samples in preventing the thermal inactivation of α-Gls, 0.2 unit/mL (16.5 nM) of this enzyme was incubated with and without 0.05 mg.mL^-1^ (2.5 μM) of wild-type and D109A αB-crystallins at 46°C. The enzyme activity was studied using an ELx 808 ELISA reader after 0, 5, 10, 15, 20, 25 and 30 minutes of incubation. The related enzyme activity was measured using p-nitrophenyl α-D-glucoside as substrate (OD = 405 nm). To evaluate the refolding activity of αB-crystallin, the enzyme (80 unit/ml) was incubated with 8 M urea in 100 mM phosphate buffer (containing 20 mM DTT, 1 mM EDTA, pH 7.0) for 90 minutes. To reach the proper final concentration of 12 μM, the incubated solution was diluted 100-fold in phosphate buffer with the presence of αB-crystallin (260 nM) and without adding the chaperone protein. For this assay, the enzyme activity was measured for 60 minutes in 10 minutes’ intervals [22, 36, 37]. The procedure of evaluating the enzyme activity was just as mentioned above.

### Cell functional analysis by *in vivo* chaperone activity assessment

The *in vivo* chaperone activity was assessed by measuring the cell survival response of the bacteria-producing either wild-type or mutant αB-crystallin following a protocol reported in our previous study [22].

### Estimation of hydrophobic index

The surface hydrophobicity in the environment of substituted amino acid was estimated using the Kyte-Doolittle hydrophobicity scale in ProtScale at ExPASY server (http://web.expasy.org/protscale).

### Statistical analyses

Data were statistically analysed by two-way ANOVA using GraphPad Prism 6.0 software. Statistical significance among the groups was determined using analyses of variance, and p < 0.05 was considered significant.

## Results

### Human αB-crystallin undergoes structural alteration due to the pathogenic D109A mutation

The validity of the created mutation (D109A) in the intended position was confirmed by DNA sequencing (S1A Fig). The solubility of wild-type and D109A mutant proteins and their purity were examined by SDS-PAGE analysis (S1B and S1C Fig). As indicated in S1C Fig, both proteins were soluble and only a small fraction of them appeared in the pellet. Subsequently, the supernatant of these proteins was used for their purification. The effect of this mutation on the intrinsic fluorescence emission of human αB-crystallin was tested at the physiological temperature (37 °C), as well as at 27 °C and 47 °C.

According to the data in S2 Fig, increasing the temperature reduces the fluorescence emission of both aromatic residues, Tyr and Trp, in the mutant protein and in its wild-type protein counterpart. The temperature-dependent intensity reduction in their fluorescence emission spectra suggests the exposure of tyrosine and tryptophan residues to the aqueous environment occurring as a consequence of thermal denaturation. Only at some temperatures, as indicated in S2 Fig, there is a slight difference in the fluorescence emission intensity of these two proteins. In fact, this finding suggests a minimal structural difference between the wild-type and mutant proteins. The synchronous fluorescence spectra further indicated the occurrence of similar structural events as a result of this mutation.

Also, the increased ANS fluorescence intensity of the mutant protein indicates both structural changes and an increase in the solvent-exposed hydrophobic surface compared to the native protein (S3A Fig). Using Kyte-Doolittle hydropathy plot, we also predicted that the hydrophobicity of the protein sequence is locally increased at the site of this mutation (S3B Fig).

**Fig 2:**
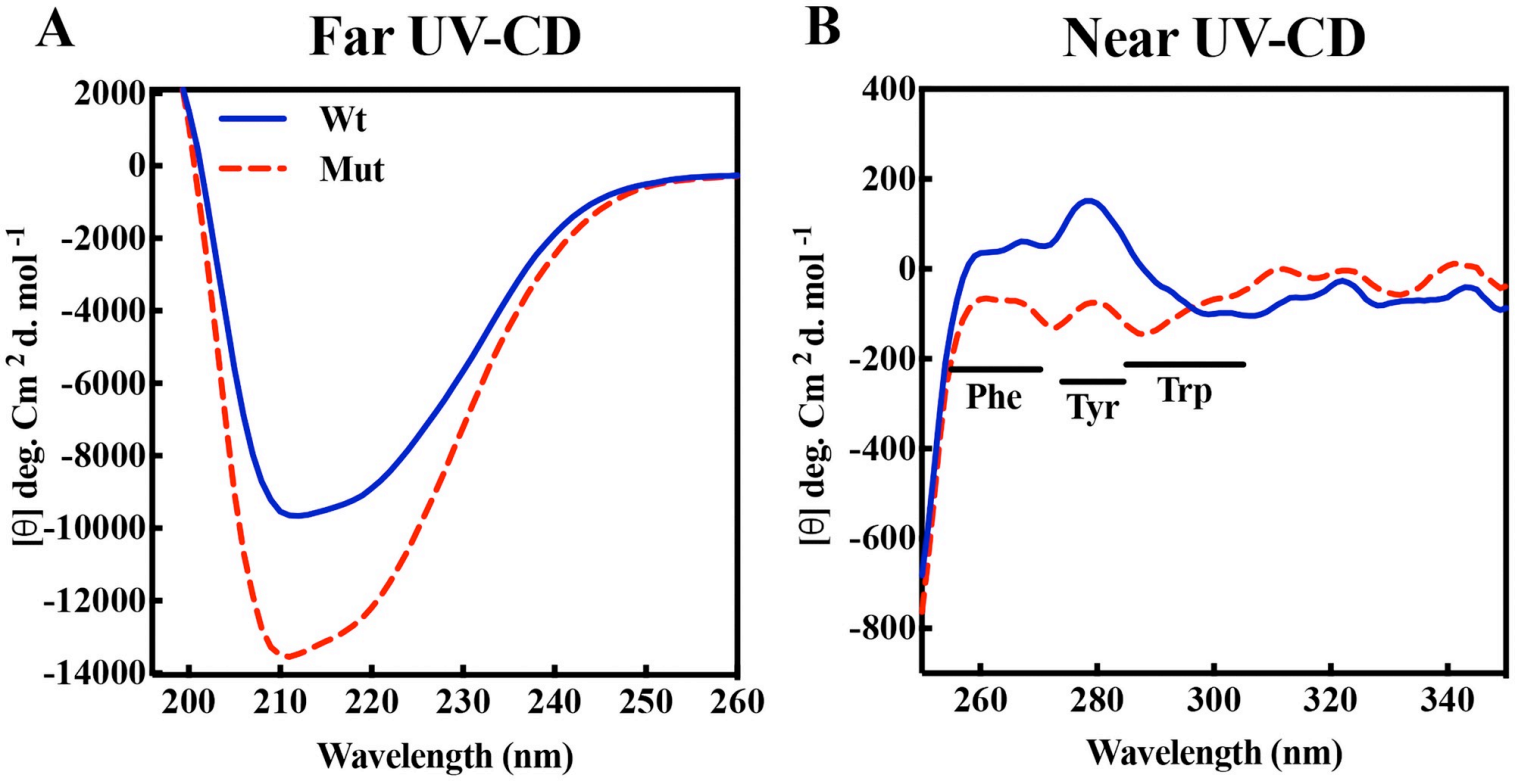
The secondary and tertiary structure studies of different αB-crystallins using UV-CD spectroscopy. **A**) Far UV-CD and **B**) Near UV-CD spectra of wild-type and mutant αB-crystallins were done in buffer A at 25 °C.

The CD analyses were also applied to assess the tertiary structural alteration of human αB-crystallin as a result of D109A mutation. As shown in Fig 2B, while the related ellipticity at 295 nm, characteristic of Trp residue, shows no difference between the two proteins, this mutation has an effect on the protein folding around Phe and Tyr residues.

The NIR spectroscopy analysis was also suggested some degree of structural changes in human αB-crystallin after D109A mutation (S4 Fig). Overall, various fluorescence, CD and NIR studies suggest that D109A mutation causes slight changes in the tertiary structure of human αB-crystallin.

Additionally, CD analyses in the far region were applied to estimate the recombinant protein secondary structural contents (Fig 2A). Quantitative analysis of the far UV-CD spectra, as indicated in Table 1, suggests that this mutation causes an evident reduction in the content of the β-sheet structure along with its conversion mainly to α-helix and to a lesser extent to the other secondary structures.

**Table 1:** The percentage of secondary structure content of different αB-crystallins obtained by the CD studies.

αB-crystallin	α-helix	β-sheet	β-turn	Random coil
wild-type	12.4 ± 0.07	33.4 ± 0.07	19.0	35.2 ± 0.08
D109A	15.4 ± 0.06	28.1 ± 0.05	19.8 ± 0.01	36.6 ± 0.06

**Fig 3:**
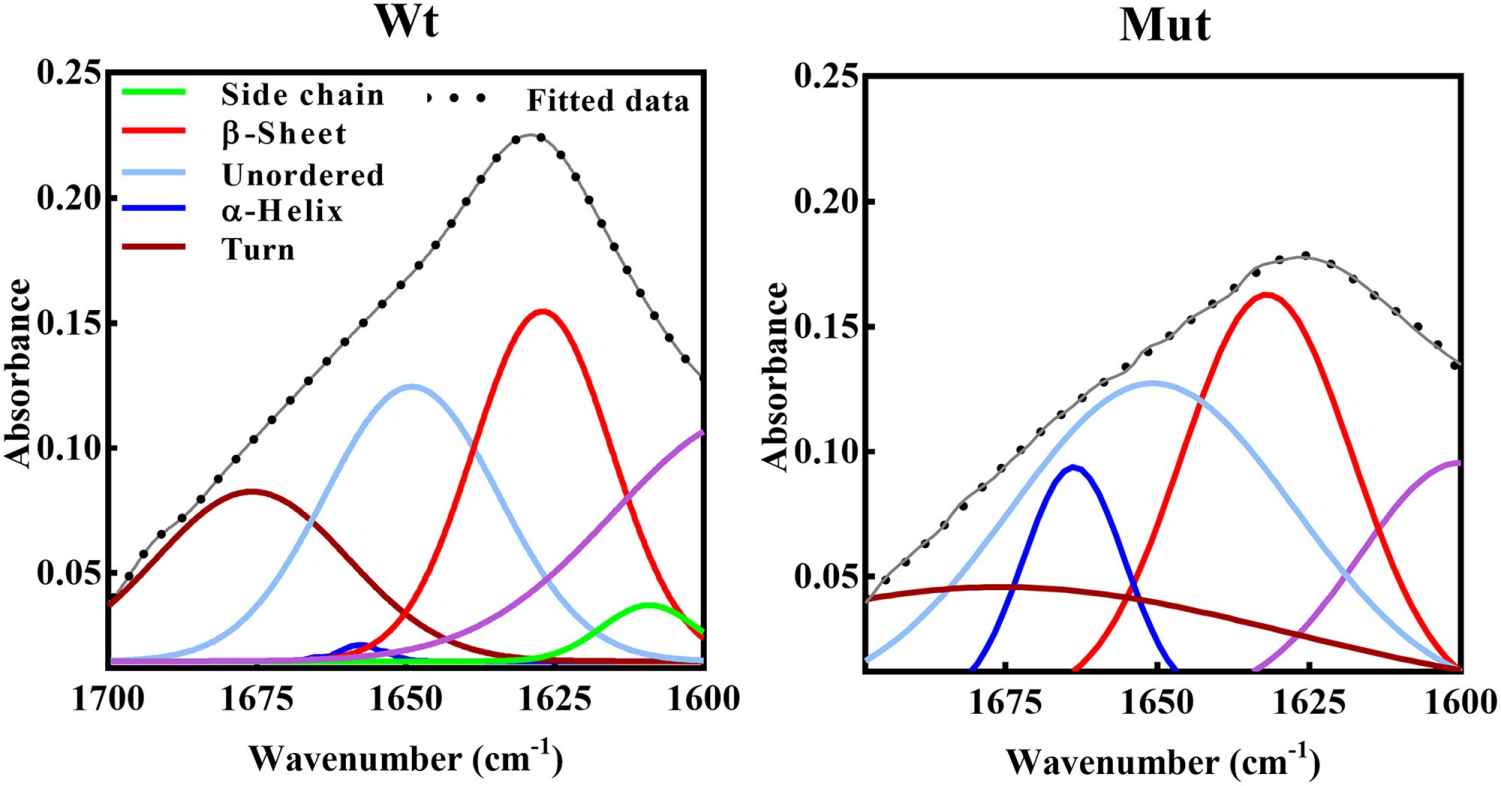
Protein secondary structural analysis using FTIR deconvoluted spectra. Wild-type and D109A mutant αB-crystallins spectra were recorded in the range of 1700–1600 cm^-1^ with a resolution of 4 cm^-1^.

FTIR as another absorption technique was also applied to further study the protein secondary structures. In our study, the FTIR analysis in the region of amide band 1 was used (Fig 3), and the amount of secondary structures presented in Table 2.

**Table 2:** The percentage of secondary structure content of different αB-crystallins obtained by FTIR analysis.

	Side chain		β-Sheet		Unordered		α-Helix		Turn	
αB-crystallin	Peak	Area	Peak	Area	Peak	Area	Peak	Area	Peak	Area
	(cm^-1^)	%	(cm^-1^)	%	(cm^-1^)	%	(cm^-1^)	%	(cm^-1^)	%
wild-type	1609	11	1627	37.1	1649	32	1658	6.1	1676	24.8
D109A	-	-	1632	31.1	1650	37	1663	10.7	1678	21.2

According to Table 2 (results of FTIR analysis), the decrease in β-sheet content of D109A αB-crystallin is accompanied by an increase in the amount of α-helix in the mutant protein.

**Fig 4:**
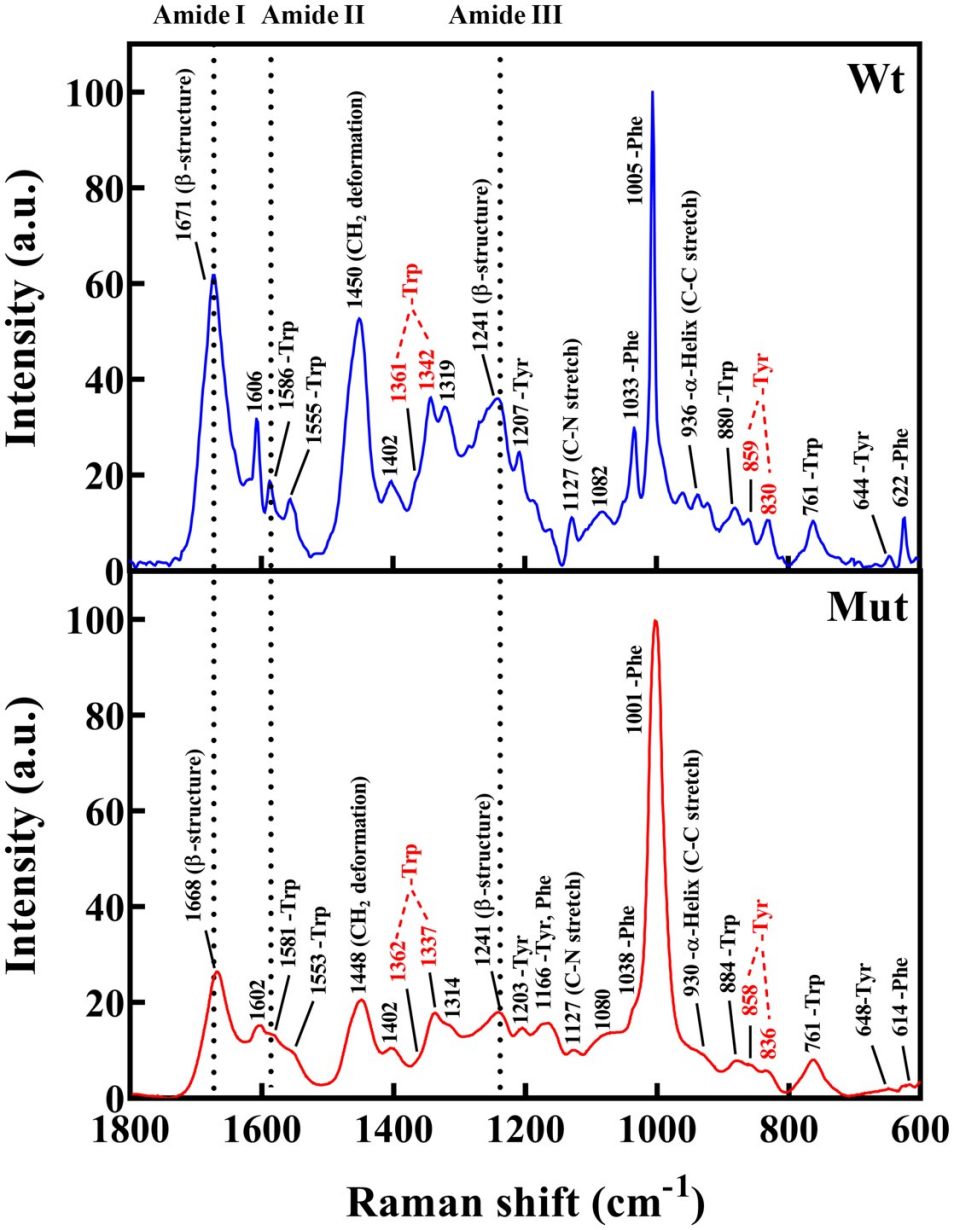
Raman spectra of human wild-type and D109A mutant αB-crystallin proteins. Similar to FTIR study, the curve fitting and deconvolution analysis of Raman spectra in amide I region was carried out and the secondary structural contents of αB-crystallin proteins were obtained. The results of both Raman and FTIR studies exhibited a reduction in β-sheet and turn contents of the D109A mutant protein, as well as the enhancement in the α-helical and unordered structures when compared with the secondary structural contents of wild-type protein counterpart (S5 Fig) [23].

The Raman spectra of D109A mutant protein and wild-type counterpart in the 1800–600 cm^-1^ region were also collected (Fig 4).

Overall, with a slight difference that can be attributed to the sample preparation methods, types of instrument used and applied deconvolution methods, the data obtained by these three methods of the secondary structural assessments largely confirm each other.

The environment around aromatic residues can also be studied by evaluation of Trp doublet (1360/1340 cm^-1^), Tyr doublet (850/830 cm^-1^), Phe (624 cm^-1^), Tyr (644 cm^-1^) and Trp (757 cm^-1^) [38, 39].

The Fermi doublet intensity ratio of Trp residue remained largely unchanged upon the D109A mutation (0.46) in human αB-crystallin (0.55). These values suggested a hydrophilic environment for this residue in the protein structure (I_1360_/I_1340_ < 1). The Tyr Fermi doublet intensity ratio (I_850_/I_830_) of wild-type and D109A mutant proteins was 0.98 and 1.2, respectively. This ratio can be influenced by the hydrogen bonding state of the Tyr phenolic hydroxyl group, and it is varying from 0.3 (phenolic OH is a strong hydrogen bond donor) to 2.5 (strong hydrogen acceptor) [38, 39]. According to the obtained ratio, D109A mutant αB-crystallin displayed a different hydrogen binding state when compared with the wild-type protein.

### The D109A mutation severely disrupts the chaperone-like activity of human αB-crystallin

**Fig 5:**
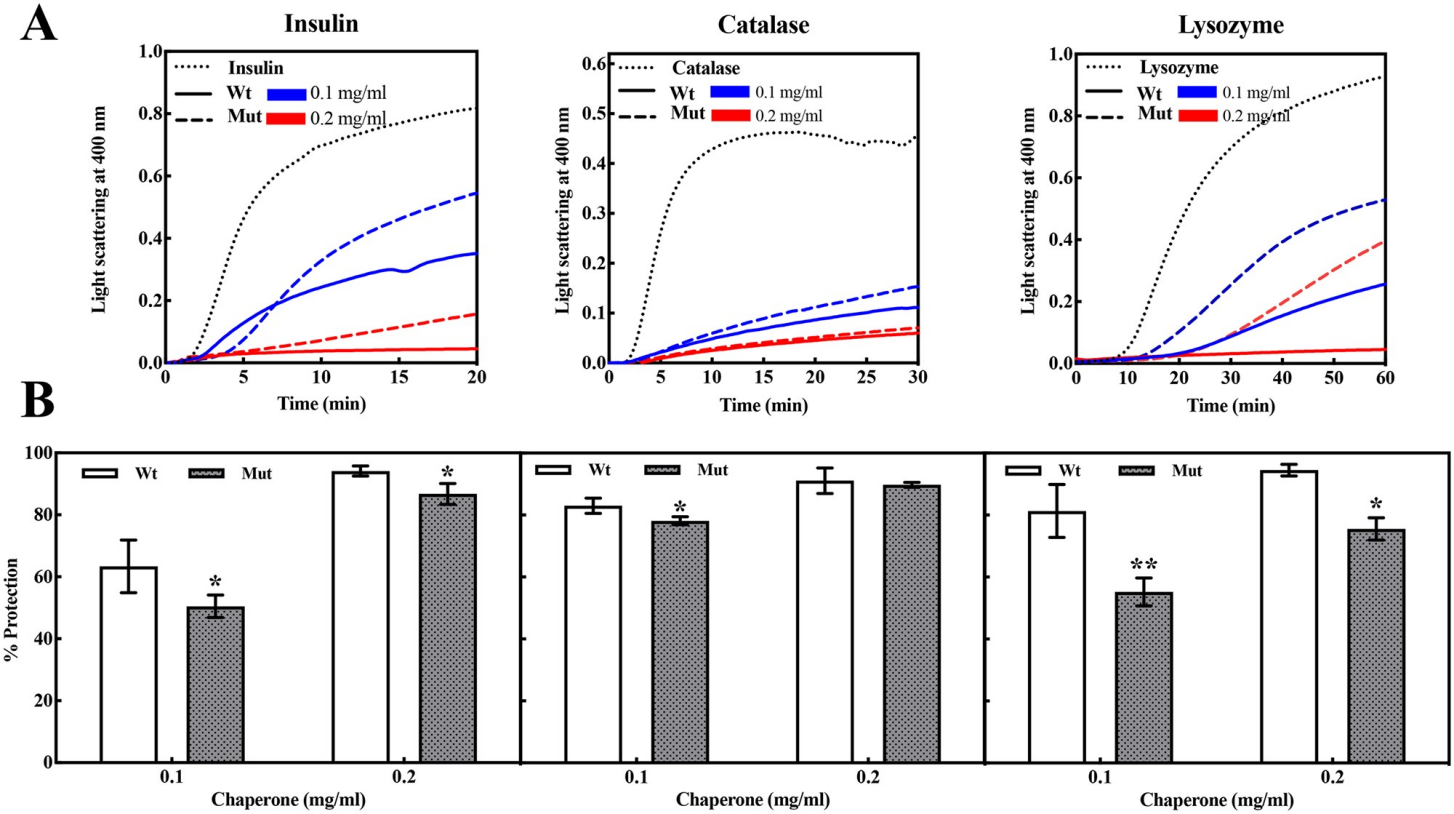
Chaperone-like activity assessment of wild-type and D109A αB-crystallins using three different target proteins. **A**) The aggregation of insulin (0.3 mg.mL^-1^) and lysozyme (0.2 mg.mL^-1^) was induced in the presence of 20 mM of DTT, at 40 °C, while the aggregation of catalase (0.3 mg.mL^-1^) was initiated at 60 °C. The light scattering spectra were recorded at 400 nm in the presence and absence of 0.1 mg.mL^-1^ and 0.2 mg.mL^-1^ of wild-type and D109A αB-crystallins, respectively. **B**) The bars represent the SD of three independent repeats and the significant difference in the percentage of protection which is marked with star (* p < 0.05, ** p < 0.01, *** p < 0.001).

As the most important activity known for the lens α-crystallin protein, the chaperone power of the mutant protein was tested in comparison with its wild-type counterpart. The anti-aggregation ability was studied in the presence of different client proteins. The percentage of protection was reported as a ratio of area under the aggregation curve of each client protein in the presence of the chaperones to the similar area in their absence (Fig 5).

**Fig 6:**
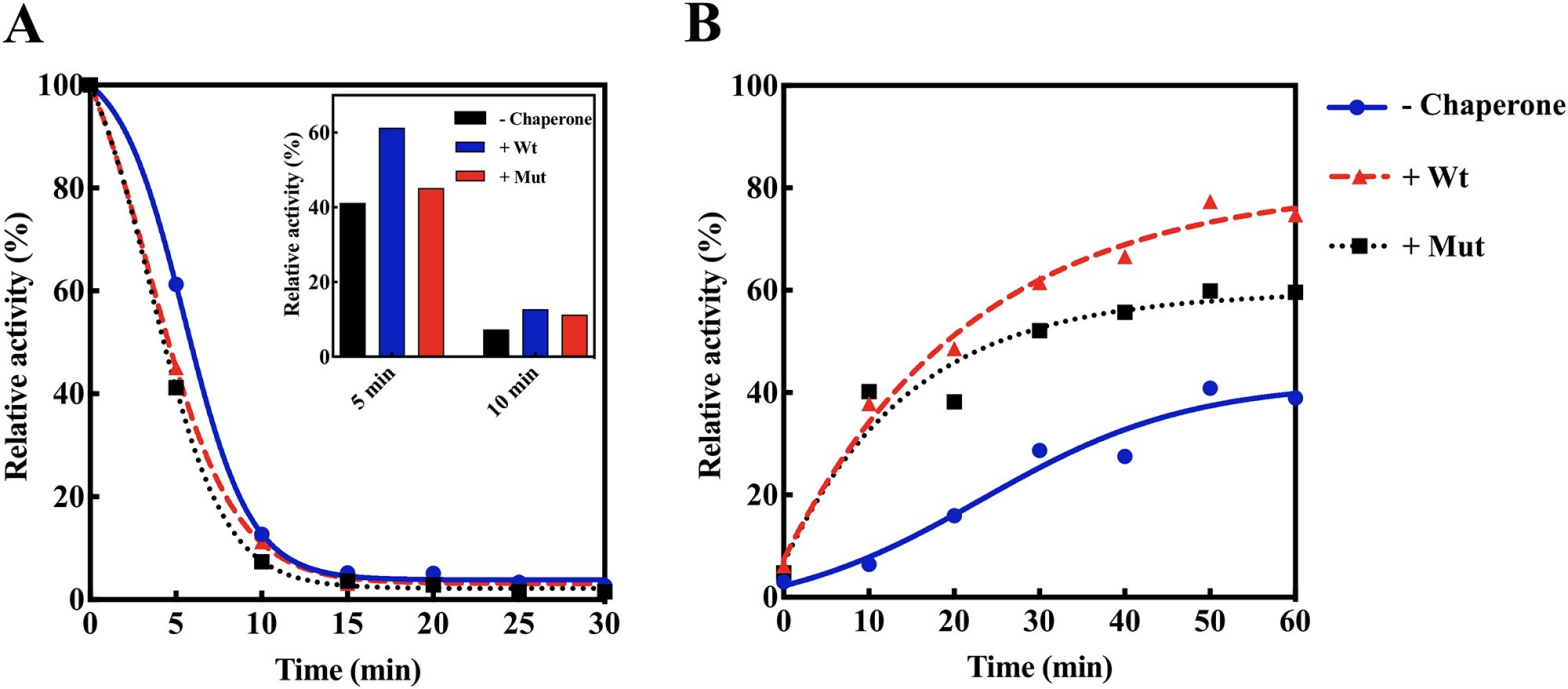
Restoring enzyme activity and refolding ability of different αB-crystallin samples against thermal and urea induced denaturation of α-Gls. **A**) The enzyme activity of α-Gls at 45 °C in the presence and absence of wild-type and D109A αB-crystallins. Thermal unfolding and inactivation of α-Gls was assessed by measuring the α-Gls activity for 30 minutes with 5 minutes interval. The inset histogram shows the relative enzyme activity at 5 and 10 minutes. **B**) The refolding of α-Gls occurred after its incubation in the refolding buffer (containing urea 8 M) at 25 °C in the presence and absence of the chaperones. The enzyme activity was measured for 60 minutes with a 10-minute interval between each evaluation.

As indicated in this figure, a remarkable difference between wild-type and D109A mutant protein in protecting aggregation of insulin and lysozyme was seen. However, there is no significant difference in their activity when catalase was used as the client protein. This finding can be explained by the dependence of the chaperone-like activity of αB-crystallin on the type of its substrate (client) protein. Another important example of chaperone activity is the ability of chaperone to maintain the activity of a target protein or enzyme under environmental stress. To test this chaperone ability, the half inactivated α-Gls after 15 min at 46 °C, was used as the target enzyme. The abilities of mutant and wild-type αB-crystallin proteins on protecting α-Gls against thermal inactivation are shown as Fig 6A.

As indicated in this figure, the mutant protein revealed a lower ability to maintain the enzyme activity of α-Gls under thermal stress. This finding, in agreement with the anti-aggregation ability of the mutant protein, indicates that D109A mutation significantly reduces chaperone activity of human αB-crystallin.

Chaperones such as α-crystallin have the ability to participate in the refolding of other proteins. Therefore, in the current study, the refolding of α-Gls enzyme in the presence of D109A mutant and wild-type proteins was investigated and compared. As shown in Fig 6B, after incubation for one hour with the refolding buffer in the absence of chaperone, α-Gls shows an activity of 39% of its original level. While after adding the wild-type and mutant chaperones, the activity of this enzyme was increased to 74.8% and 59.6%, respectively. The results of this study clearly suggested that the refolding ability, as one of the most important examples of chaperone activity, in human αB-crystallin is greatly reduced by this pathogenic mutation.

**Fig 7:**
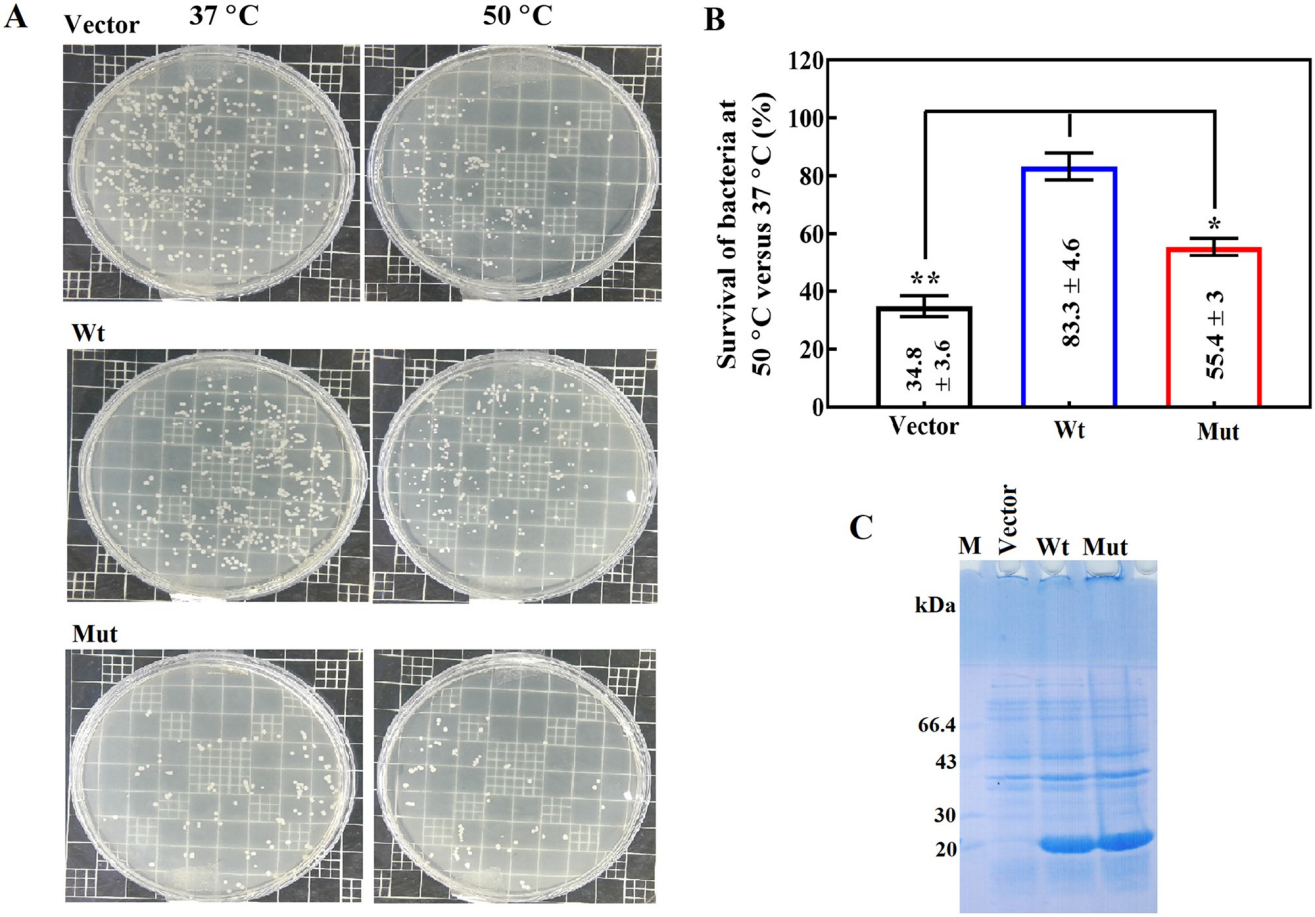
Effect of heat stress on the survival of *Escherichia coli* expressing the chaperone proteins. **A)** This figure indicates the colonies formed at 37 °C and 50 °C. **B)** This figure shows the survival of *Escherichia coli* cells by measuring the ratio of colonies formed at 37 °C and 50 °C. The values are reported as averages of three independent repeats (* p <0.05, ** p <0.01, *** p <0.001). **C**) SDS-PAGE gel shows a similar expression level of the wild-type and mutant αB-crystallin proteins.

The effect of D109A mutation on the survival of the bacterial host cells was investigated under heat stress (Fig 7). The effect of chaperone on the survival of the host cells was determined as an indicator of the cell function by counting the number of colonies and determining their ratio at 50 °C to 37 °C.

Compared to the cells bearing basal vector, the bacteria expressing wild-type αB-crystallin indicated a significantly higher survival ability under the heat shock. Also, the D109A mutant αB-crystallin was indicated a weaker survival effect compared to the wild-type protein counterpart. As shown in Fig 7C, the changes in the survival response at 50 °C are not related to variation in the expression level of these proteins in the host cells (Fig 7C). Overall, the results of our study suggested that various bioactivities related to the chaperone action of this ocular lens protective protein may also be damaged by the occurrence of D109A mutation.

### The D109A mutant protein shows a larger oligomer sizes compared to the wild-type protein counterpart

**Fig 8:**
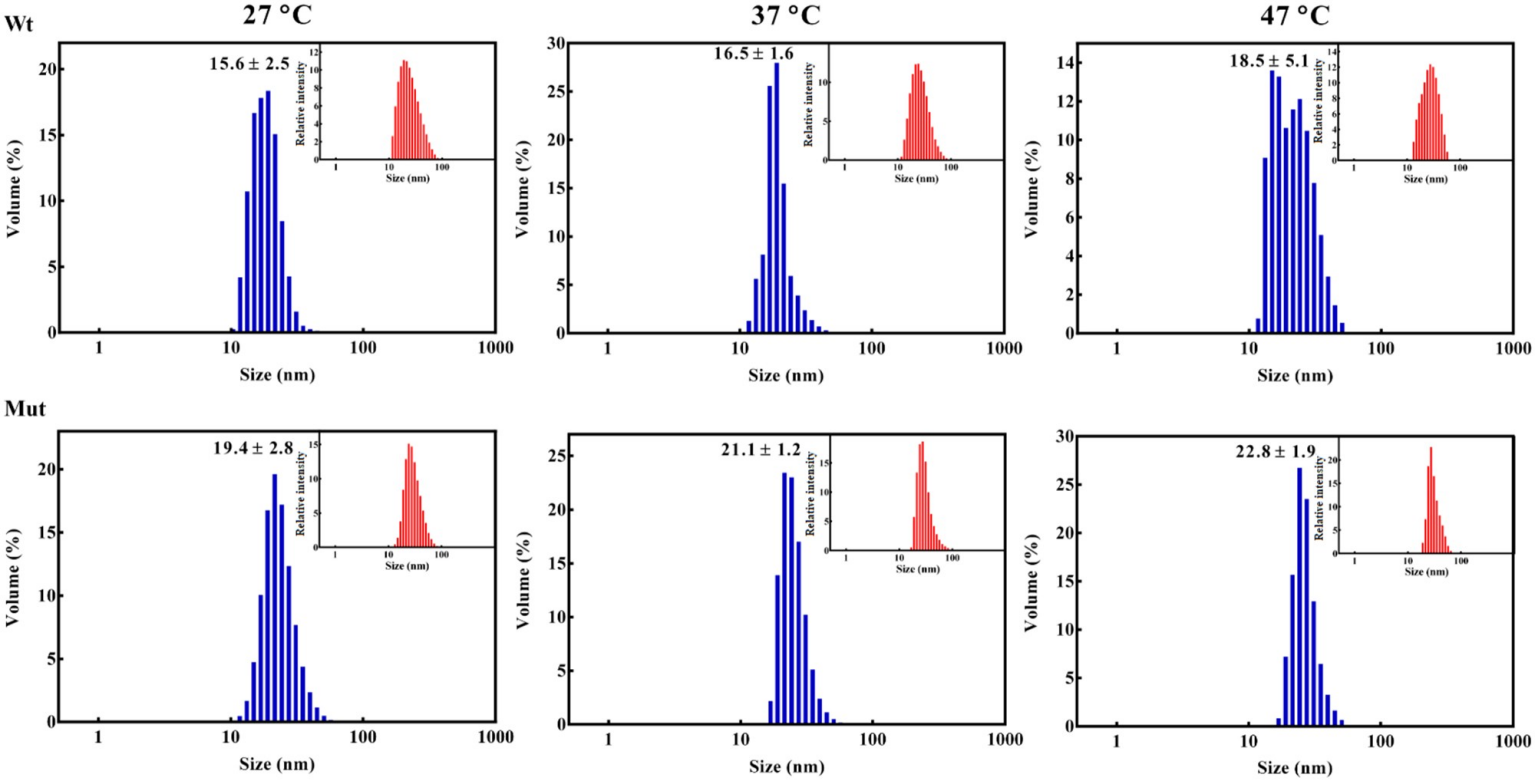
Study of the oligomerization state of different αB-crystallin samples. Dynamic light scattering studies on the protein samples (1 mg. mL^-1^) prepared in buffer A, at three different temperatures. The relative intensity versus size is shown as the inset figures.

In this study, both DLS and analytical ultracentrifugation (AUC) were used to study the size of oligomers in the mutated protein. The DLS studies revealed that the size of the oligomers in D109A αB-crystallin is significantly larger than that of wild-type protein counterpart (Fig 8). Moreover, the DLS assessments suggested that both proteins show a slight increase in the oligomeric size distribution with the temperature elevation. However, at all three different temperatures the mutant protein has a larger size distribution than the wild-type protein counterpart (Fig 8).

**Fig 9:**
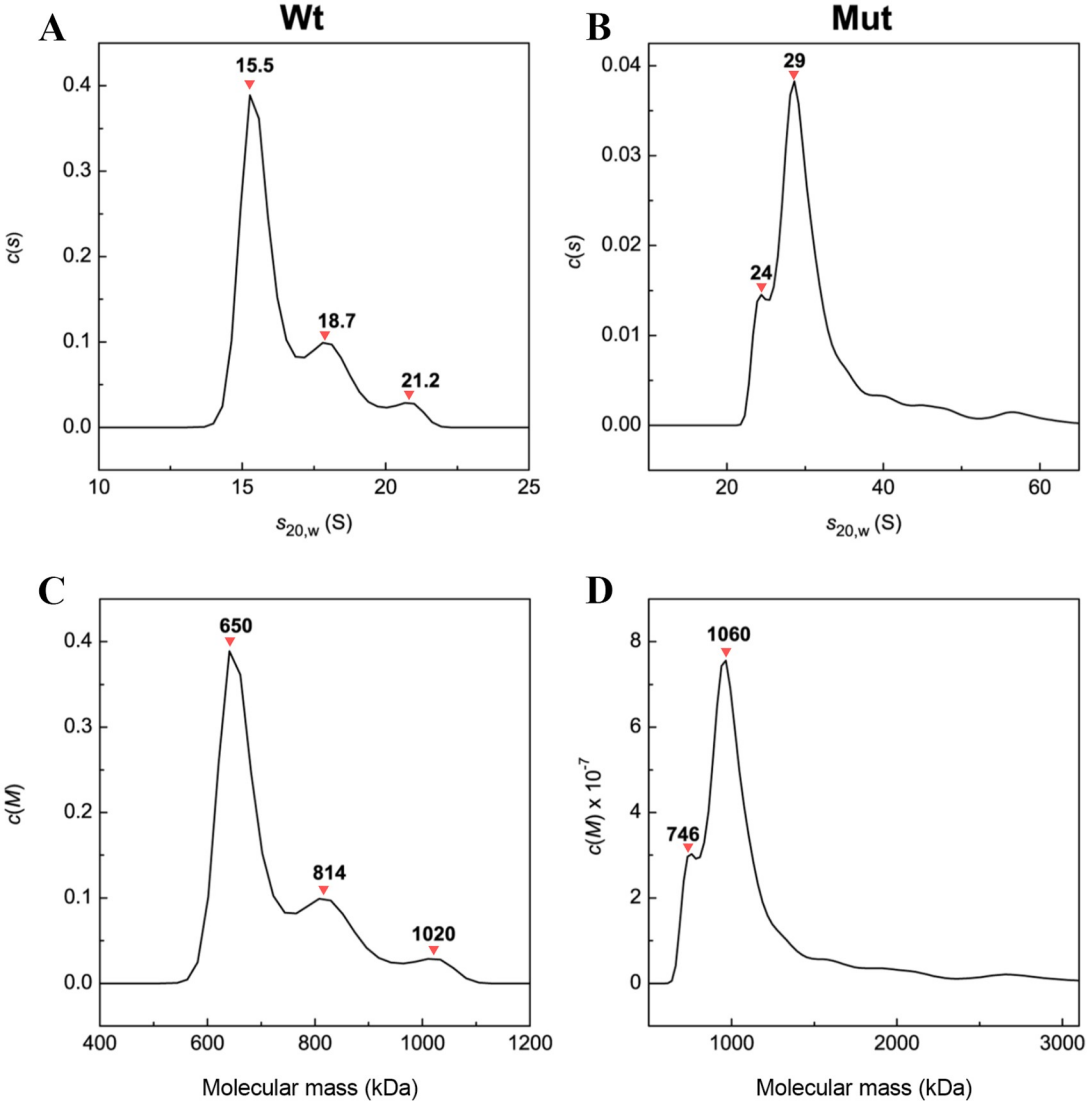
Analytical ultracentrifugation (AUC) analyses of different human αB-crystallin samples. Differential sedimentation coefficient distributions *c*(*s*) (**A** and **B**) and differential mass distribution *c*(*M*) (**C** and **D**) for αB-crystallin and D109A mutant at 20 °C. Rotor speed was 40,000 rpm.

The larger sedimentation coefficient (S) of the mutant oligomers (two populations of 29 S and 24 S) in comparison with the wild-type oligomers (three populations of 15.5 S, 18.7 S and 21.2 S) was also observed during the AUC analyses (Fig 9).

In agreement with the DLS study, the AUC assessment also suggested larger molecular mass for the mutant protein oligomers. As indicated in the comparison of *c*(*s*) distributions between D109A mutant and wild-type αB-crystallins in panels A and B (Fig 9), the mutation largely affects this distribution. Also, the *c*(*s*) distribution is shifted towards the larger sedimentation coefficient values (*s*_20, W_) in the region of 20–60 S (Fig 9B). This observation indicates that the proportion of large oligomers in the *c*(*s*) distribution increases. It is also seen from the comparison of the molecular mass distributions *c*(*M*) obtained from the *c*(*s*) distributions (Fig 9A and 9B) as presented in panels C and D. The majority of oligomers of the wild-type and mutant proteins were appeared about 650 and 1,060 kDa in size (Fig 9C and 9D). Additionally, according to the *c*(*M*) versus molecular mass plots, the number of subunits in the oligomers of wild-type and D109A mutant protein was calculated to be 32 and 53, respectively (Fig 9C and 9D). Also, there are higher molecular weight polydisperse ensembles with a mass in the range of 1500–3000 kDa in panel D. It should be also noted that after 24 hours of incubation of samples at 20 °C the distribution of *c*(*s*) for wild-type αB-crystallin becomes more compact with two populations of oligomers (16.3 and 19.2 S) and an average sedimentation coefficient of (17.7 ± 2.0) S. At the same time, the distribution for the mutant protein becomes more polydisperse with an average sedimentation coefficient of (34.0 ± 4.6) S, which corresponds to significantly larger oligomers. Based on the results of AUC study, it is suggested that D109A mutation shifts the equilibrium between the oligomer and dimer states to the larger protein mass, which in turn can participate in scattering of the light entering to the eye lenses.

### The D109A mutation reduces the conformational stability of human αB-crystallin

**Fig 10:**
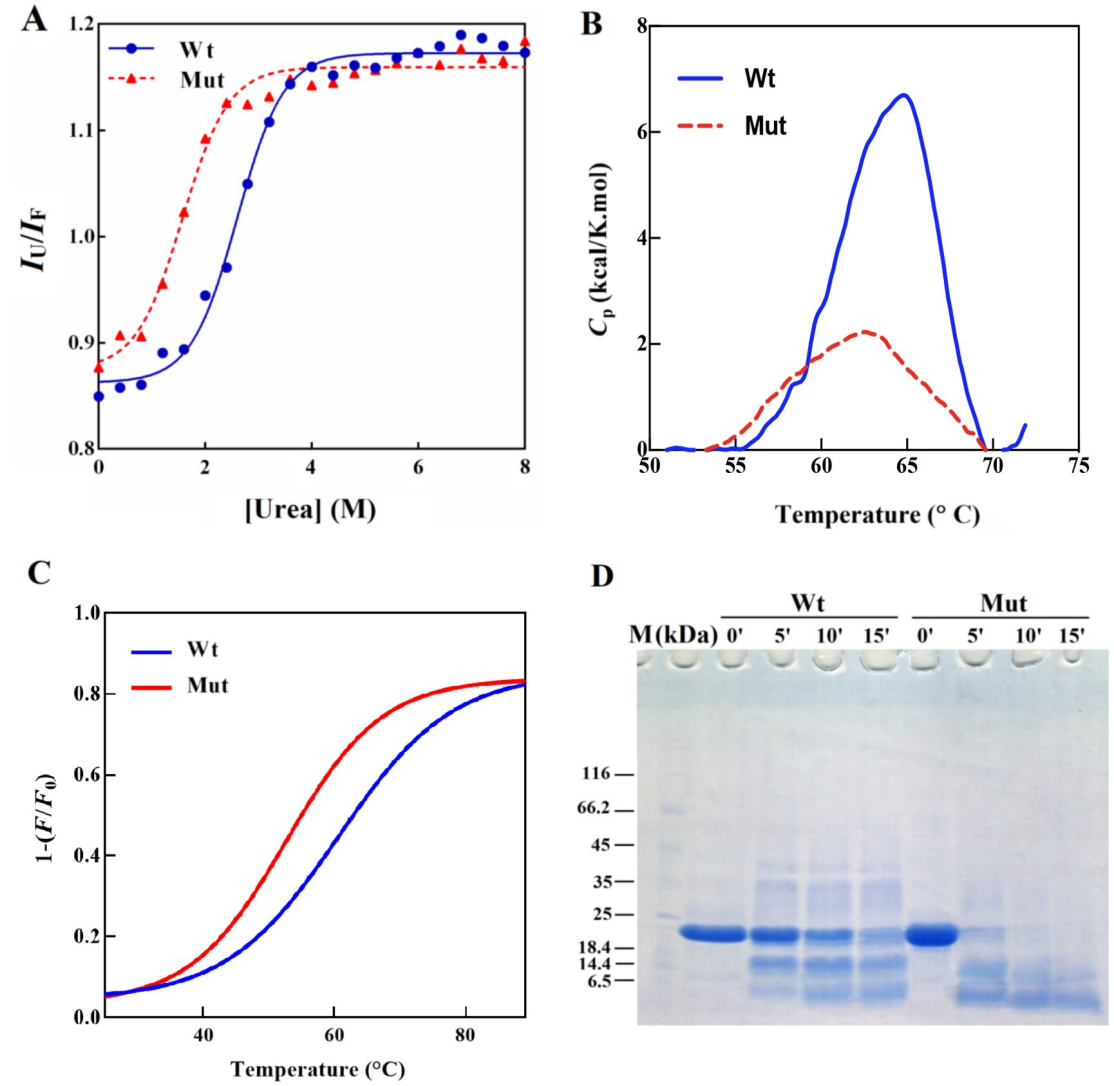
The stability assessment of D109A mutant and wild-type human αB-crystallins. **A**) The stability of protein samples against chemical denaturation. Protein samples were incubated with an increasing concentration of urea (0–8 M) for 18 hours and the tryptophan fluorescence spectra of all samples were recorded. **B**) Thermal-stability of human αB-crystallins was measured by DSC. The plots display the heat capacity changes (Δ*C*_p_) of the different αB-crystallin proteins against various temperatures. **C**) Thermal stability assessment of αB-crystallin proteins by Intrinsic Trp fluorescence assay. **D**) The stability of protein samples against the proteolytic activity of α-chymotrypsin. The protein samples were incubated with the protease for 5, 10 and 15 minutes at 37 °C.

In the present study, the chemical stability of the mutant protein was studied and compared to the wild-type protein counterpart in the presence of urea denaturing agent (Fig 10A and Table 3).

**Table 3:** Δ*G*^0^ and *C*_1/2_ values of different αB-crystallin samples which obtained from the equilibrium urea unfolding assessment.

αB-crystallin	Δ*G*^0^ (kcal/mol)	*C*_1/2_ (M)
**wild-type**	6.09 ± 0.99	2.61 ± 0.24
**D109A**	3.76 ± 0.81*	1.41 ± 0.33

* p < 0.05,

** p < 0.01,

*** p < 0.001

As shown, denaturation of half of the mutant proteins occurs at the lower concentrations of urea. Moreover, Δ*G*° of chemical unfolding of D109A protein was lower than that of the wild-type protein counterpart (Table 3). The results of this study suggest that due to this mutation, the chemical stability of human αB-crystallin is reduced by about half.

To further investigate the stability of D109A αB-crystallin, the heat capacity change (Δ*C*_p_) of wild-type and D109A mutant αB-crystallin was plotted under thermal unfolding condition (Fig 10B). The thermodynamic parameters, Δ*H* and Δ*S*, were also calculated, using Eqs. (2)-(5) and presented as Table 4. The area under each curve is equal to Δ*H* (overall enthalpy change), which is a manifestation of the protein denaturation [34]. As shown in Fig 10B, the wild-type αB-crystallin displayed unfolding transition midpoints (*T*_m_) at 64.8°C when D109A mutant protein indicated a lower value of *T*_m_ (62.5°C) compared to the wild-type αB-crystallin, as well as the Δ*H* and Δ*S* parameters [23, 40, 41]. Also, the Trp thermal unfolding fluorescence profile indicated similar results to DSC analysis. However, the difference in unfolding temperature between wild-type (*T*_m_ = 61°C) and mutant (*T*_m_ = 52.6°C) proteins was larger than that obtained by DSC. As indicated in Fig 10C, the *T*_m_ of the mutant protein is significantly lower compared to the wild-type protein, showing that the mutant protein is less stable than its wild-type protein counterpart.

**Table 4:** Thermodynamic parameters of αB-crystallin protein samples by DSC assessment.

αB-crystallin	Δ*H* (kcal/mol)	Δ*S* (kcal/K·mol)	*T*_m_ (°C)
**wild-type**	11.9 ± 0.08	0.035 ± 0.003	64.8 ± 0.07
**D109A**	5.1 ± 0.12	0.015 ± 0.001	62.5 ± 0.06

In addition to the chemical and thermal stabilities, the proteolytic stability of the mutant protein was investigated in the presence of chymotrypsin. Our results suggested an important decrease in the proteolytic stability of the mutant protein in comparison to the wild-type protein (Fig 10D). This result is in accordance with the obtained results of the chemical denaturation studies as described above (Fig 10A–10C). Overall, this mutation has an important conformational destabilizing effect on human αB-crystallin and this instability may play a role in the pathogenesis of the mutant protein.

### The D109A mutation alters the amyloidogenic feature of human αB-crystallin

Fibrillation of the mutant and wild-type proteins was studied under thermal stress. For this purpose, in addition to the ThT fluorescence spectroscopic method, two microscopic approaches including fluorescence microscopy and transmission electron microscopy (TEM) were applied.

**Fig 11:**
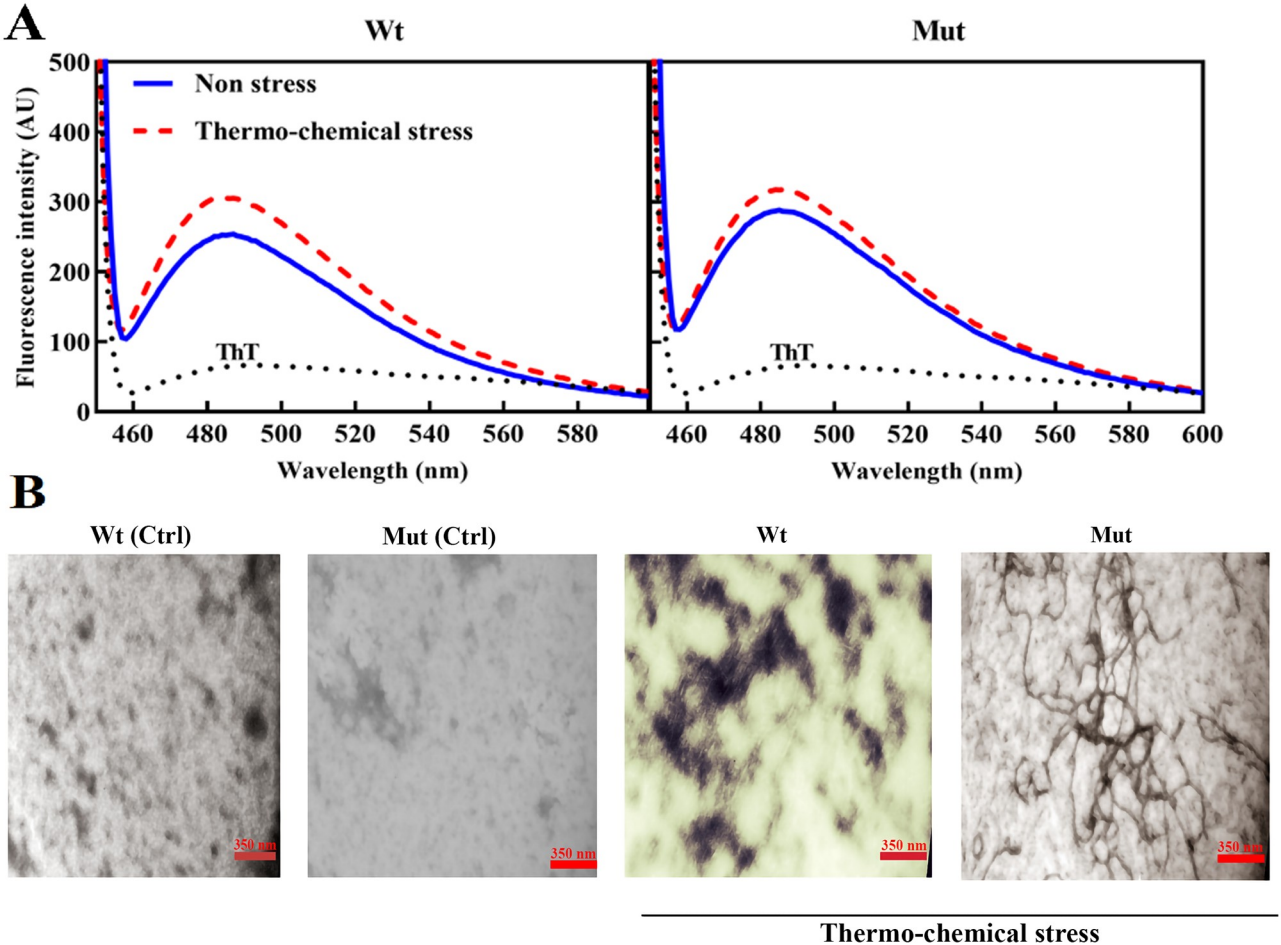
Comparing amyloidogenic properties of D109A and wild-type αB-crystallins before and after thermochemical stress. The results of ThT fluorescence assay (**A**) and TEM analysis (**B**) are indicated in this figure.

The protein samples were incubated for 4 days at 60°C in the presence of 1 M guanidine hydrochloride. The ThT fluorescence study is an important method for studying the formation of protein amyloid fibrils, but sometimes its increase may be related to the rise of only beta-sheet structural content in protein without fibril formation. Our data suggested an increase in the ThT fluorescence intensity of both wild-type and mutant proteins after the thermal stress (Fig 11A). However, the increase in ThT fluorescence emission of the protein samples after incubation under thermochemical stress was not large enough to accurately attributed to the formation of amyloid fibril as measured by this method. Protein aggregate formation was also studied in the presence of ThT under a fluorescence microscope. The results of this study also made it clear that thermochemical stress causes the formation of a significant amount of the protein aggregates in both native and mutant proteins (shown as see S6 Fig). The formation of the protein amyloid fibrils was also studied by TEM analyses (Fig 11B). The results of TEM analysis suggested that both wild-type and mutant proteins were capable of forming amyloid fibrils under thermochemical stress but as shown in Fig 11B the mutated protein has more propensity to form fibril.

## Discussion

αB-crystallin is expressed not only in the eye lenses but also in many other tissues such as brain, heart, kidney and striated muscles [8, 11, 12]. Some mutations in this chaperone protein indicate an important association with development of cataract, myofibrillar myopathy (MFM) or both [15–19]. MFM is a muscular dystrophic disorder that primarily affects the skeletal muscles moving the body and, in some cases, causes important problems with the cardiac muscle [42]. Recently, an important mutation in the human αB-crystallin gene (*CRYAB*) has been reported that, in addition to being involved in cataracts, it contributes to the development of cardiomyopathy [21]. This mutation (D109A) occurs in an evolutionarily conserved α-crystallin domain (ACD), playing a central role in structure and chaperone-like activity of the guardian protein of eye lenses (α-crystallin) [6, 21]. This mutation replaces a charged-polar residue (aspartate) with a relatively small non-polar amino acid (alanine) that at physiological pH has no negative charge. Under the physiological conditions, aspartate is also able to participate in the electrostatic interactions and hydrogen bonding while alanine lacks such important features.

Therefore, D109A mutation is expected to have an important structural and functional consequences in human αB-crystallin, which further explains its pathogenic role in development of cataracts and cardiac myopathy. In the continuation of this section, we will try to clarify how this mutation may cause the above-mentioned pathological states. As results of performing D109A mutation, important changes were observed in the secondary structure (as shown by CD, Raman, FTIR assessments), tertiary structure (as indicated by fluorescence, CD, Raman studies) and quaternary structure (as revealed by AUC and DLS) of human αB-crystallin which may alter the strength and quality of its fine interactions with different target proteins in the lenticular and muscular tissues [43–46]. It is well known that a flexible dynamic structure is required for the functioning of αB-crystallin. The equilibrium between oligomeric forms is very sensitive to changes in the cellular environment and crowding conditions [47, 48]. In the eye lenses, there is a very delicate pattern of interactions among α-crystallin and its other natural partners, such as beta- and gamma-crystallin [49], so that as a result of the structural alteration, a slightest change in these interactions is expected to cause important optical focusing problem, leading to development of the visual perturbation [43–46]. In a similar way, in muscular tissues, by causing significant structural changes as indicated in this study, this mutation probably affects the interaction of human αB-crystallin with its significant target protein (desmin), providing the basis for development of the cardiomyopathy (Scheme 1).

**Scheme 1. The pathomechanism underlying cataract and cardiomyopathy development by D109A mutant αB-crystallin.** As a result of D109A mutation, the native structure of human αB-crystallin undergoes important changes that subsequently affect the interactions of this protein with desmin and other intermediate filaments [43–46]. The end result of these changes is a decrease in its cytoprotective ability in the cardiac muscle cells, resulting in the development of cardiomyopathy [14, 19, 18, 44, 50]. Also, an important decrease in chaperone-like activity along with the formation of large oligomers [16, 44, 45] that scatter light explains the role of the D109A mutant protein in the occurrence of the cataract disorder.

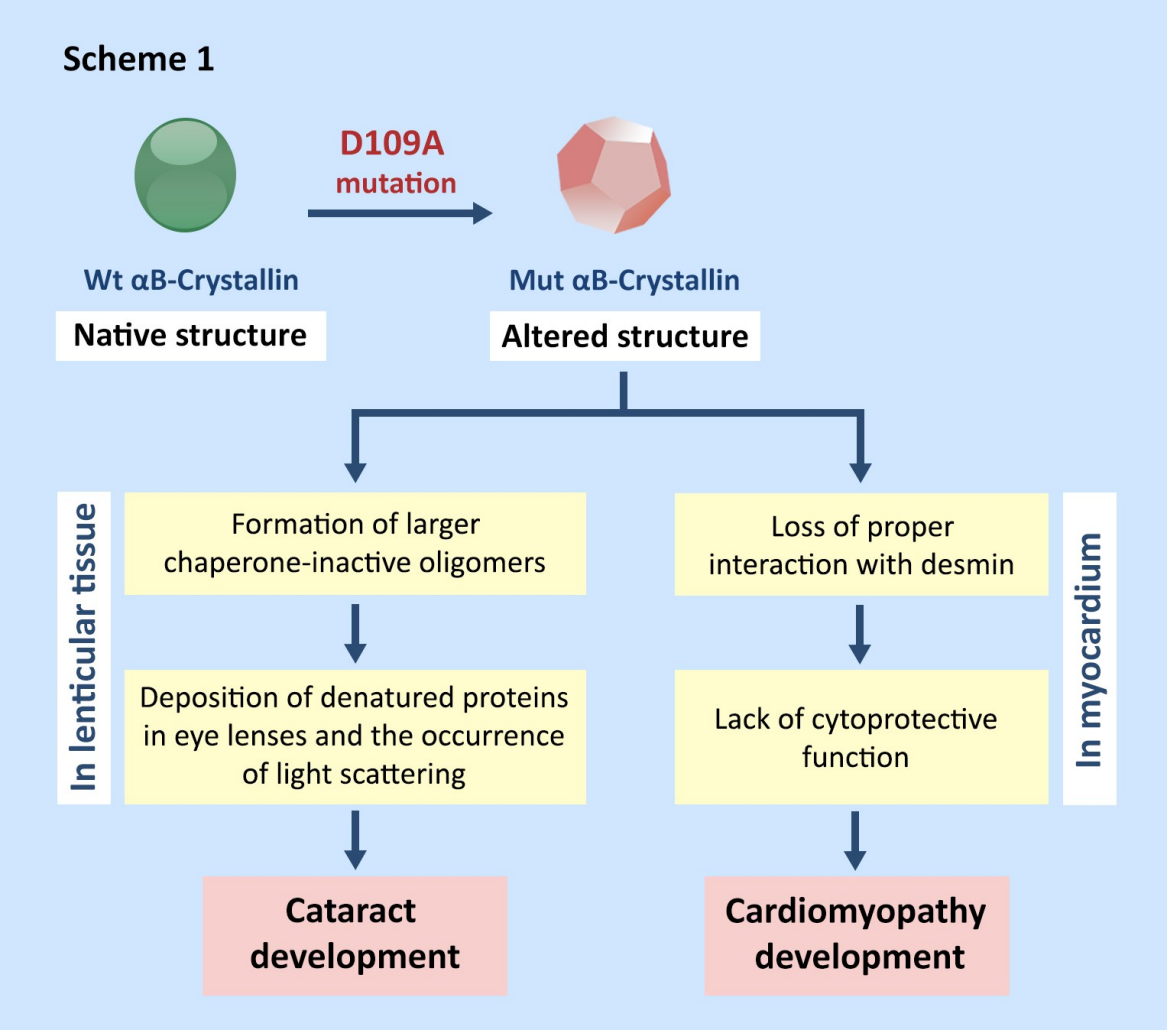


Decreased chaperone-like activity of this protein due to D109A mutation (Fig 5) largely reduces the ability of human αB-crystallin to prevent the deposition of other denatured proteins in the lenticular tissues and provides the basis for development of opacity and cataracts in the eye lenses. Also, the tendency of the mutant αB-crystallin to form larger oligomeric masses (Figs 8 and 9) could potentially scatter light entering the eye lenses and reduce the quality of vision.

According to the results of the structural studies it can be suggested that the main difference between the folding of the wild-type and D109A mutant proteins is not happening at micro environmental levels (S2 Fig) but at the secondary structures (Figs 2A, 3 and 4, S4 and S5 Figs, Tables 1 and 2), leading to tertiary structural alteration of the mutant protein (Figs 2B and 4) which subsequently affects its solvent-exposed hydrophobic surface (S3 Fig). The increase in the hydrophobic patches of the mutant protein (S3A Fig) may also explain the larger size of oligomers in this protein as observed by DLS and AUC studies (Figs 8 and 9).

It can also be suggested that the structural alterations in D109A mutant protein will result in the easier and further exposure of α-chymotrypsin cleavage sites, leading to its easier digestion by this protease (Fig 10D). The lack of stability against proteolytic activity may be a result of the instability in the overall structure of D109A human αB-crystallin as demonstrated by an important decrease in Δ*G*^0^ and *C*_1/2_ (Fig 10A, Table 3). Moreover, it can be estimated that the enhanced amyloidogenic properties of D109A αB-crystallin are because of the lack of stability in its structure (Fig 11). The more hydrophobic nature of the mutant protein (S3 Fig) can be considered as one of the important conditions facilitating its easer formation of amyloid fibrils (Fig 11). The different chaperone-like activity of mutant and wild-type proteins in the presence of each client protein is because of their substrate-dependent chaperoning function (Fig 5). It is also obvious that our chaperone molecules have shown their best activity in the presence of catalase as a target protein. This observation is due to the thermal-induced aggregation of catalase improves the chaperone-like activity of these proteins under thermal stress (Fig 5). The poor anti-aggregation ability, reduced refolding power and the attenuated ability in restoring enzyme activity under thermal stress by D109A mutant αB-crystallin in comparison to the wild-type protein counterpart (Figs 5 and 6) are perhaps linked to the structural alteration and ability of the mutant protein for formation of larger oligomers. This suggestion may come true because α-crystallin is in a state of equilibrium between the chaperone-active dimers and the chaperone-inactive oligomers [51], and any factor that shifts this equilibrium to the oligomeric state reduces its chaperone power.

## Conclusion

The results of our study in different ways suggested that the substitution of conserved aspartate residue at position 109 in the primary structure of human αB-crystallin with alanine has a serious consequence on the higher structural levels, from secondary to quaternary structures of the mutated protein. Moreover, this mutation significantly reduces stability and chaperone-like activity of the mutant protein and increases its tendency for both oligomerization and aggregation. Overall, it can be suggested that the pathogenicity of D109A mutant αB-crystallin has important links with its attenuated chaperone-like activity, less stable nature, formation of the larger oligomers and the higher tendency to form amyloid fibrils under the thermochemical stress condition.

## Supporting information

S1 Fig Site-directed mutagenesis and protein purification studies. **A**) The D109A and wild-type αB-crystallins cDNA sequencing results. **B**) The SDS-PAGE profile of purified mutant and wild-type human αB- crystallins. **C**) The solubility of the proteins was evaluated by SDS-PAGE analysis. Mut and Wt respectively stand for the mutant and wild-type proteins (S: supernatant, P: pellet).
(TIF)

S2 Fig The fluorescence spectra of wild-type and mutant αB-crystallins. Tyr, Trp and synchronous fluorescence spectra of the protein samples (0.15 mg.mL^-1^) at 27 °C, 37 °C and 47 °C were collected. The excitation wavelength was set at 280 nm for Tyr spectrum (**A**) and 295 nm for Trp spectrum (**B**). The emission spectra were obtained between 300 and 500 nm. **C**) The excitation wavelength in synchronous study for Tyr and Trp was set at 15 nm and 60 nm, respectively and the emission spectra were scanned between 200 and 350 nm.
(TIF)

S3 Fig The hydrophobicity assessment of wild-type and D109A αB-crystallins. **A**) The surface hydrophobicity was studied using ANS probe. The protein samples with a concentration of 0.15 mg. mL^-1^ were excited at 365 nm, while the emission spectra were collected between 400–600 nm. **B**) The estimation of the changes in the hydrophobicity of human αB-crystallin around D109A mutation using the Expasy server of Protoscale software.
(TIF)

S4 Fig NIR spectroscopic analysis of the protein powders. The spectra were recorded at 25 °C in the range of 8,000 to 4,000 cm^-1^ with a resolution of 8 cm^-1^. Mut and Wt respectively stand for the mutant and wild-type proteins.
(TIF)

S5 Fig Deconvolution analysis of Raman spectra and comparative calculation of the secondary structural contents of different αB-Cry proteins. **A**) Curve fitting and deconvolution analysis of Raman spectra in amide I region were carried out using the peaks as described in FTIR method section. **B**) Comparison of the secondary structural content of different protein samples by FTIR and Raman spectroscopy. Mut and Wt respectively stand for the mutant and wild-type proteins.
(TIF)

S6 Fig The amyloidogenic properties study of D109A and wild-type human αB-crystallin by fluorescence microscopy. Protein samples (2 mg.mL^-1^ prepared in buffer A) were incubated at 60 °C for 4 days and after a 5 min incubation of each protein sample (0.15 mg.mL^-1^) with 20 μM ThT. The fluorescence microscopy studies were used to analyse the fiber plaques of protein samples using the green filter (GFP) with an excitation and emission wavelength of 469 nm and 525 nm, respectively. Mut and Wt respectively stand for the mutant and wild-type proteins.
(TIF)

S1 Raw images (ZIP)

S1 Graphical abstract (TIF)

## References

[1] Gusev NB, Bogatcheva NV, Marston SB. Structure and properties of small heat shock proteins (sHsp) and their interaction with cytoskeleton proteins. Biochemistry (Mosc.). 2002; 67: 511–9. 12059769 10.1023/a:1015549725819

[2] MacRae TH. Structure and function of small heat shock/α-crystallin proteins: established concepts and emerging ideas. Cell Mol Life Sci. 2000; 57: 899–913. doi: 10.1007/pl00000733 10950306 PMC11146919

[3] de Jong WW, Caspers GJ, Leunissen JAM. Genealogy of the alpha-crystallin-small heat-shock protein superfamily. Int J Biol Marcomol. 1998; 22: 151–62. doi: 10.1016/s0141-8130(98)00013-0 9650070

[4] Horwitz J. α-Crystallin can function as a molecular chaperone. Proc Natl Acad Sci U S A 1992; 89: 10449–53. doi: 10.1073/pnas.89.21.10449 1438232 PMC50356

[5] Dubin RA, Ally AH, Chung S, Piatigorsky J. Human alpha B-crystallin gene and preferential promoter function in lens. Genomics. 1990; 7: 594–601. doi: 10.1016/0888-7543(90)90204-8 2387586

[6] Horwitz J. Alpha-crystallin. Exp Eye Res. 2003; 76: 145–53. doi: 10.1016/s0014-4835(02)00278-6 12565801

[7] Van der Ouderaa FJ, De Jong WW, Bloemendal H. The amino acid sequence of the alpha A2 chain of bovine alpha crystalline. Eur J Biochem. 1973; 39: 207–22. doi: 10.1111/j.1432-1033.1973.tb03119.x 4770792

[8] Taylor RP, Benjamin IJ. Small heat shock proteins: a new classification scheme in mammals. J Mol Cell Cardiol. 2005; 38: 433–44. doi: 10.1016/j.yjmcc.2004.12.014 15733903

[9] Reddy GB, Kumar PA, Kumar MS. Chaperone‐like activity and hydrophobicity of α‐crystallin. IUBMB life. 2006; 58: 632–641. doi: 10.1080/15216540601010096 17085382

[10] Bloemendal H, de Jong W, Jaenicke R, Lubsen N, Slingsby C, Tardieu A. Ageing and vision: structure, stability and function of lens crystallins. Prog Biophys Mol Biol. 2004; 86: 407–85. doi: 10.1016/j.pbiomolbio.2003.11.012 15302206

[11] Longoni S, James P, Chiesi M. Cardiac α-crystallin. I. Isolation and identification. Mol Cell Biochem. 1990; 99: 113–20. 2291764

[12] Dubin R, Wawrousek E, Piatigorsky J. Expression of the murine αB-crystallin gene is not restricted to the lens. Mol Cell Biol. 1989; 9: 1083–91. doi: 10.1128/mcb.9.3.1083-1091.1989 2725488 PMC362698

[13] Graw J. Genetics of crystallins: cataract and beyond. Exp Eye Res. 2009; 88: 173–89. doi: 10.1016/j.exer.2008.10.011 19007775

[14] Sacconi S, Feasson L, Antoine JC, Pecheux C, Bernard R, Cobo AM, et al. A novel CRYAB mutation resulting in multisystemic disease. Neuromuscul Disord. 2012; 22: 66–72. doi: 10.1016/j.nmd.2011.07.004 21920752

[15] Chen Q, Ma J, Yan M, Mothobi ME, Liu Y, Zheng F. A novel mutation in *CRYAB* associated with autosomal dominant congenital nuclear cataract in a Chinese family. Mol Vis. 2009; 15: 1359–65. 19597569 PMC2709425

[16] Li H, Li C, Lu Q, Su T, Ke T, Li DWC, et al. Cataract mutation P20S of alpha B-crystallin impairs chaperone activity of alpha A-crystallin and induces apoptosis of human lens epithelial cells. Biochim Biophys Acta. 2008; 1782: 303–9. doi: 10.1016/j.bbadis.2008.01.011 18343237

[17] Khan AO, Abu Safieh L, Alkuraya FS. Later retinal degeneration following childhood surgical aphakia in a family with recessive CRYAB mutation (p.R56W). Ophthalmic Genet. 2010; 31: 30–6. doi: 10.3109/13816810903452047 20141356

[18] Vicart P, Caron A, Guicheney P, Li Z, Prévost MC, Faure A, et al. A missense mutation in the αB-crystallin chaperone gene causes a desmin-related myopathy. Nat Genet. 1998; 20: 92–5. doi: 10.1038/1765 9731540

[19] Raju I, Abraham EC. Mutants of human αB-crystallin cause enhanced protein aggregation and apoptosis in mammalian cells: influence of co-expression of HspB1. Biochem Biophys Res Commun. 2013; 430: 107–12. doi: 10.1016/j.bbrc.2012.11.051 23194663 PMC3544980

[20] Datskevich PN, Nefedova VV, Sudnitsyna MV, Gusev NB. Mutations of small heat shock proteins and human congenital diseases. Biochemistry (Mosc.). 2012; 77: 1500–14. doi: 10.1134/S0006297912130081 23379525

[21] Fichna JP, Potulska-Chromik A, Miszta P, Redowicz MJ, Kaminska AM, Zekanowski C, et al. A novel dominant D109A *CRYAB* mutation in a family with myofibrillar myopathy affects αB-crystallin structure. BBA Clin. 2017; 7: 1–7. doi: 10.1016/j.bbacli.2016.11.004 27904835 PMC5124346

[22] Khoshaman K, Yousefi R, Tamaddon AM, Abolmaali SS, Oryan A. Moosavi-Movahedi AA, et al. The impact of different mutations at Arg54 on structure, chaperone-like activity and oligomerization state of human αA-crystallin: The pathomechanism underlying congenital cataract-causing mutations R54L, R54P and R54C. Biochim Biophys Acta- Proteins Proteomics. 2017; 1865: 604–18. doi: 10.1016/j.bbapap.2017.02.003 28179137

[23] Ghahramani M, Yousefi R, Krivandin A, Muranov K, Kurganov B, Moosavi-Movahedi AA. Structural and functional characterization of D109H and R69C mutant versions of human αB-crystallin: the biochemical pathomechanism underlying cataract and myopathy development. Int J Biol Macromol. 2019; 146: 1142–60. doi: 10.1016/j.ijbiomac.2019.09.239 31678106

[24] Sharma KK, Kumar GS, Murphy AS, Kester K. Identification of 1,1′-bi(4-anilino) naphthalene-5,5′-disulfonic acid binding sequences in alpha-Cry. J Biol Chem. 1998; 273: 15474–78. doi: 10.1074/jbc.273.25.15474 9624133

[25] Meehan S, Berry Y, Luisi B, Dobson CM, Carver JA, MacPhee CE. Amyloid fibril formation by lens crystallin proteins and its implications for cataract formation. J Biol Chem. 2004; 279: 3413–19. doi: 10.1074/jbc.M308203200 14615485

[26] Whitmore L, Wallace B.A.DICHROWEB, an online server for protein secondary structure analyses from circular dichroism spectroscopic data. Nucleic Acids Res. 2004; 32: 668–73. doi: 10.1093/nar/gkh371 15215473 PMC441509

[27] Maltesen MJ, van de Weert M, Grohganz H. Design of experiments-based monitoring of critical quality attributes for the spray-drying process of insulin by NIR spectroscopy. AAPS Pharm Sci Tech. 2012; 13: 747–55. doi: 10.1208/s12249-012-9796-1 22585372 PMC3429688

[28] Liang JJ, Chakrabarti B. Intermolecular interaction of lens crystallins: From rotationally mobile to immobile states at high protein concentrations. Biochem Biophys Res Commun. 1998; 246: 441–45. doi: 10.1006/bbrc.1998.8640 9610380

[29] Surewicz WK, Olesen PR. On the thermal stability of α-crystallin: A new insight from infrared spectroscopy. Biochemistry. 1995; 34: 9655–60. doi: 10.1021/bi00030a001 7626634

[30] Kengne-Momo RP, Daniel P, Lagarde F, Jeyachandran YL, Pilard JF, Durand-Thouand MJ, et al. Protein interactions investigated by the Raman spectroscopy for biosensor applications. Int J Spectro. 2012; 2012: 1–7.

[31] Zhou C, Qi W, Lewis EN, Carpenter JF. Concomitant Raman spectroscopy and dynamic light scattering for characterization of therapeutic proteins at high concentrations. Anal Biochem. 2015; 472: 7–20. doi: 10.1016/j.ab.2014.11.016 25475399

[32] Brown PH, Schuck P. Macromolecular size-and-shape distributions by sedimentation ultracentrifugation. Biophys J. 2006; 90: 4651–61. doi: 10.1529/biophysj.106.081372 16565040 PMC1471869

[33] Persikov AV, Xu Y, Brodsky B. Equilibrium thermal transitions of collagen model peptides. Protein Sci. 2004; 13: 893–902. doi: 10.1110/ps.03501704 15010541 PMC2280063

[34] Schon A, Velazquez-Campoy A. Calorimetry, in: Jiskoot W, Crommelin DJA (Eds.), Methods for Structural Analysis of Protein Pharmaceuticals. AAPS Press, Arlington, VA, 2005, pp. 573–589.

[35] Akbarian M, Yousefi R, Moosavi-Movahedi AA, Ahmad A, Uversky VN. Modulating insulin fibrillation using engineered B-chains with mutated C-termini. Biophys J. 2019; 117: 1626–41. doi: 10.1016/j.bpj.2019.09.022 31607389 PMC6838759

[36] Biswas A, Das KP. Alpha-crystallin assisted refolding of enzyme substrates: optimization of external parameters. Protein J. 2007; 26: 247–55. doi: 10.1007/s10930-006-9066-8 17211683

[37] Jakob U, Gaestel M, Engel K, Buchner J. Small heat shock proteins are molecular chaperones. J Biol Chem. 1993; 268: 1517–20. 8093612

[38] Rygula A, Majzner K, Marzec KM, Kaczor A, Pilarczyk M, Baranska M. Raman spectroscopy of proteins: a review. J Raman Spectrosc. 2013; 44: 1061–76.

[39] Bursell SE, Yu NT. Fluorescence and Raman spectroscopy of the crystalline lens. in: Masters BR (Ed.), Noninvasive Diagnostic Techniques in Ophthalmology. Springer, New York, NY, 1990, pp. 319–41.

[40] Michnik A. Thermal stability of bovine serum albumin DSC study. J Therm Anal Calorim. 2003, 71: 509–19.

[41] Walsh MT, Sen AC, Chakrabarti B. Micellar subunit assembly in a three-layer model of oligomeric alpha-crystallin. J Biol Chem. 1991; 266: 20079–84. 1939070

[42] Olivé M, Kley RA, Goldfarbd LG. Myofibrillar myopathies: new developments. Curr Opin Neurol. 2013; 26: 527–35. doi: 10.1097/WCO.0b013e328364d6b1 23995273 PMC5127196

[43] Liu Y, Zhang X, Luo L, Wu M, Zeng R, Cheng G, et al. A novel alphaB-crystallin mutation associated with autosomal dominant congenital lamellar cataract. Invest Ophthalmol Vis Sci. 2006; 47: 1069–75. doi: 10.1167/iovs.05-1004 16505043 PMC2078606

[44] Bova MP, Yaron O, Huang Q, Ding L, Haley DA, Stewart PL, et al. Mutation R120G in αB-crystallin, which is linked to a desmin-related myopathy, results in an irregular structure and defective chaperone-like function. Proc Natl Acad Sci U S A. 1999; 96: 6137–6142. doi: 10.1073/pnas.96.11.6137 10339554 PMC26848

[45] Perng MD, Muchowski PJ, van Den IJssel P, Wu GJ, Hutcheson AM, Clark JI, et al. The cardiomyopathy and lens cataract mutation in alphaB-crystallin alters its protein structure, chaperone activity, and interaction with intermediate filaments in vitro. J Biol Chem. 1999; 274: 33235–43. doi: 10.1074/jbc.274.47.33235 10559197

[46] Song S, Hanson MJ, Liu BF, Chylack LT, Liang JJ. Protein-protein interactions between lens vimentin and alphaB-crystallin using FRET acceptor photobleaching. Mol Vis. 2008; 14: 1282–1287. 18618007 PMC2447818

[47] Chebotareva NA, Roman SG, Borzova VA, Eronina TB, Mikhaylova VV, Kurganov BI. Chaperone-like activity of HSPB5: The effects of quaternary structure dynamics and crowding. Int J Mol Sci. 2020; 21: E4940. doi: 10.3390/ijms21144940 32668633 PMC7404038

[48] Chebotareva NA, Eronina TB, Roman SG, Mikhaylova VV, Sluchanko NN, Gusev NB, et al. Oligomeric state of alphaB-crystallin under crowded conditions. Biochem Biophys Res Commun. 2019; 508: 1101–5. doi: 10.1016/j.bbrc.2018.12.015 30551876

[49] Takemoto L, Sorensen CM. Protein-protein interactions and lens transparency. Exp Eye Res. 2008; 87: 496–501. doi: 10.1016/j.exer.2008.08.018 18835387 PMC2666974

[50] Maloyan A, Sanbe A, Osinska H, Westfall M, Robinson D, Imahashi K, et al. Mitochondrial dysfunction and apoptosis underlie the pathogenic process in alpha-B-crystallin desmin-related cardiomyopathy. Circulation. 2005; 112: 3451–61. doi: 10.1161/CIRCULATIONAHA.105.572552 16316967 PMC1398051

[51] Ecroyd H, Carver JA. Crystallin proteins and amyloid fibrils. Cell Mol Life Sci. 2009; 66: 62–81. doi: 10.1007/s00018-008-8327-4 18810322 PMC11131532

