# Supplementary figures and images for: Structural and functional studies of D109A human αB-crystallin contributing to the development of cataract and cardiomyopathy diseases
Source: PLoS One. 2021 Nov 29;16(11):e0260306. doi: 10.1371/journal.pone.0260306 (PMC8629256; doi:10.1371/journal.pone.0260306)

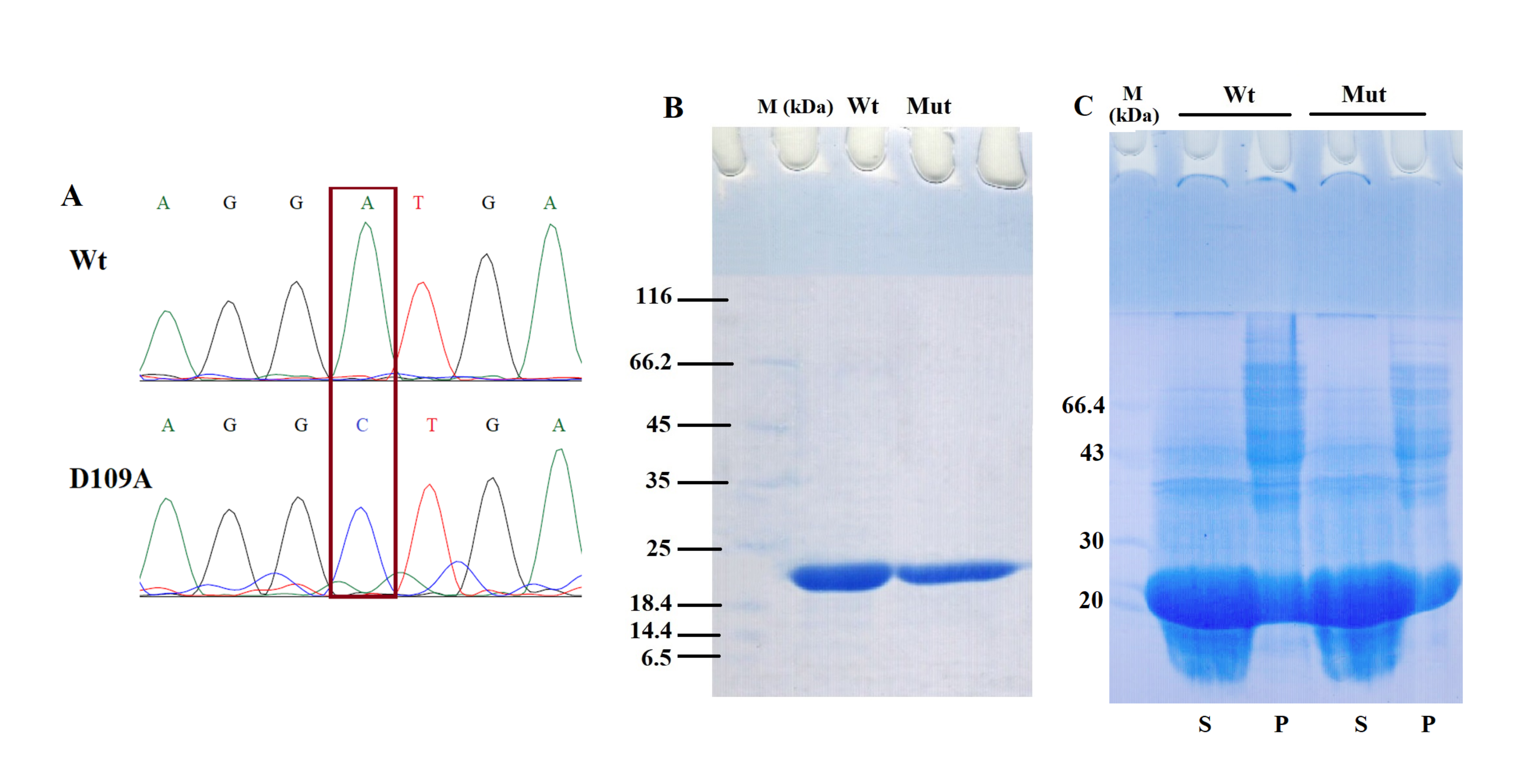

Supplement: S1 Fig — A) The D109A and wild-type αB-crystallins cDNA sequencing results. B) The SDS-PAGE profile of purified mutant and wild-type human αB- crystallins. C) The solubility of the proteins was evaluated by SDS-PAGE analysis. Mut and Wt respectively stand for the mutant and wild-type proteins (S: supernatant, P: pellet). (TIF) [file pone.0260306.s001.tif]

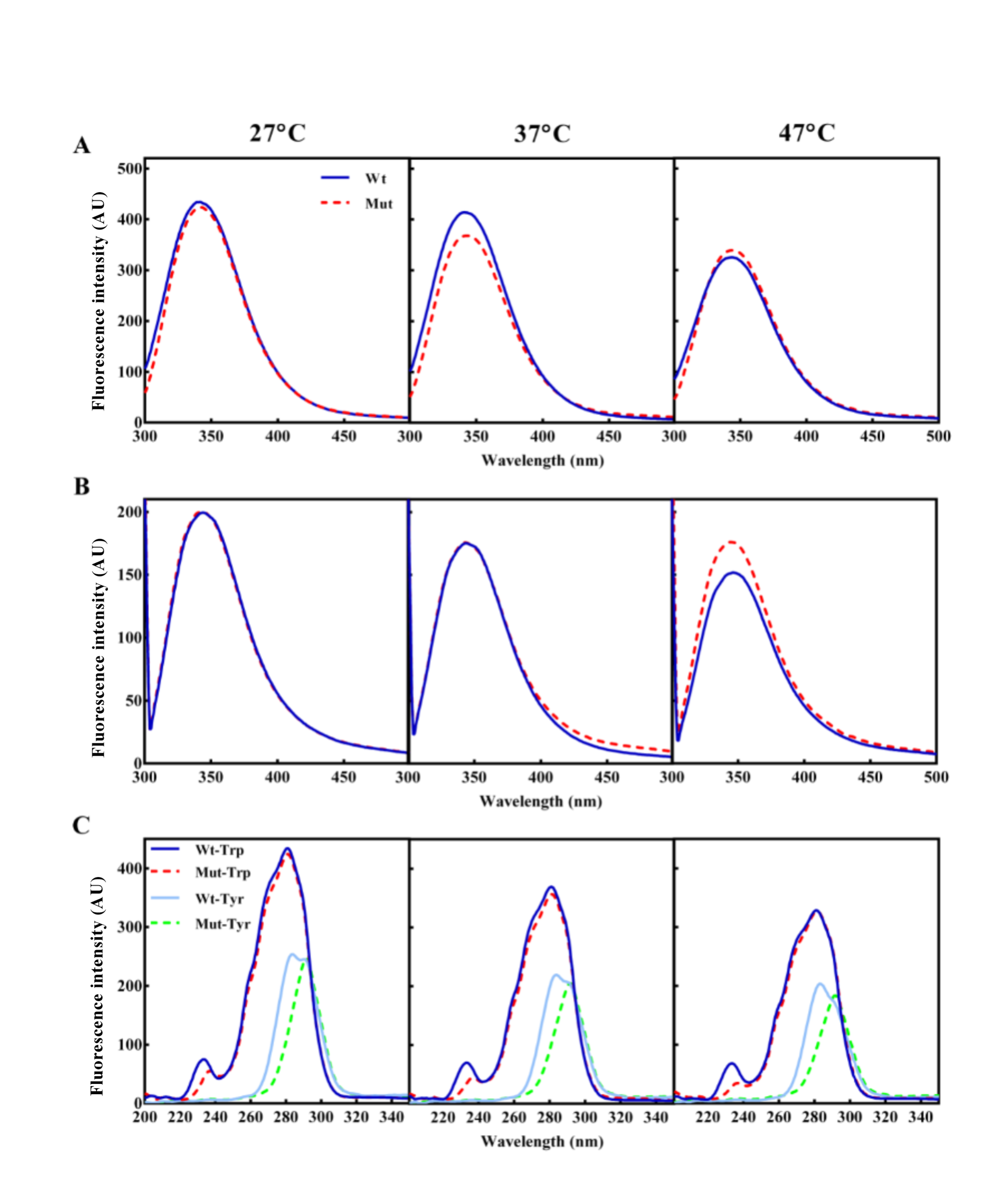

Supplement: S2 Fig — Tyr, Trp and synchronous fluorescence spectra of the protein samples (0.15 mg.mL-1) at 27 °C, 37 °C and 47 °C were collected. The excitation wavelength was set at 280 nm for Tyr spectrum (A) and 295 nm for Trp spectrum (B). The emission spectra were obtained between 300 and 500 nm. C) The excitation wavelength in synchronous study for Tyr and Trp was set at 15 nm and 60 nm, respectively and the emission spectra were scanned between 200 and 350 nm. (TIF) [file pone.0260306.s002.tif]

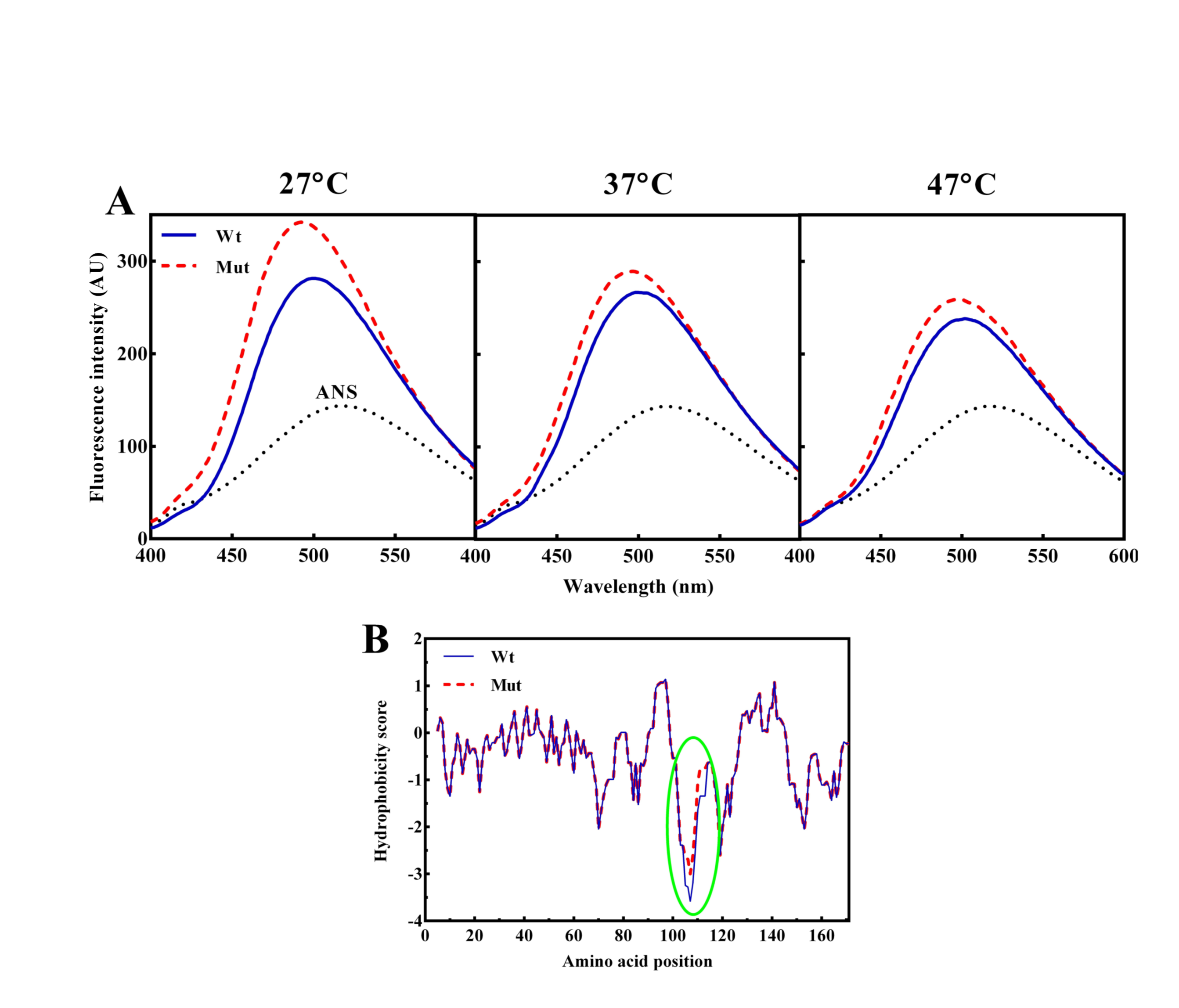

Supplement: S3 Fig — A) The surface hydrophobicity was studied using ANS probe. The protein samples with a concentration of 0.15 mg. mL-1 were excited at 365 nm, while the emission spectra were collected between 400–600 nm. B) The estimation of the changes in the hydrophobicity of human αB-crystallin around D109A mutation using the Expasy server of Protoscale software. (TIF) [file pone.0260306.s003.tif]

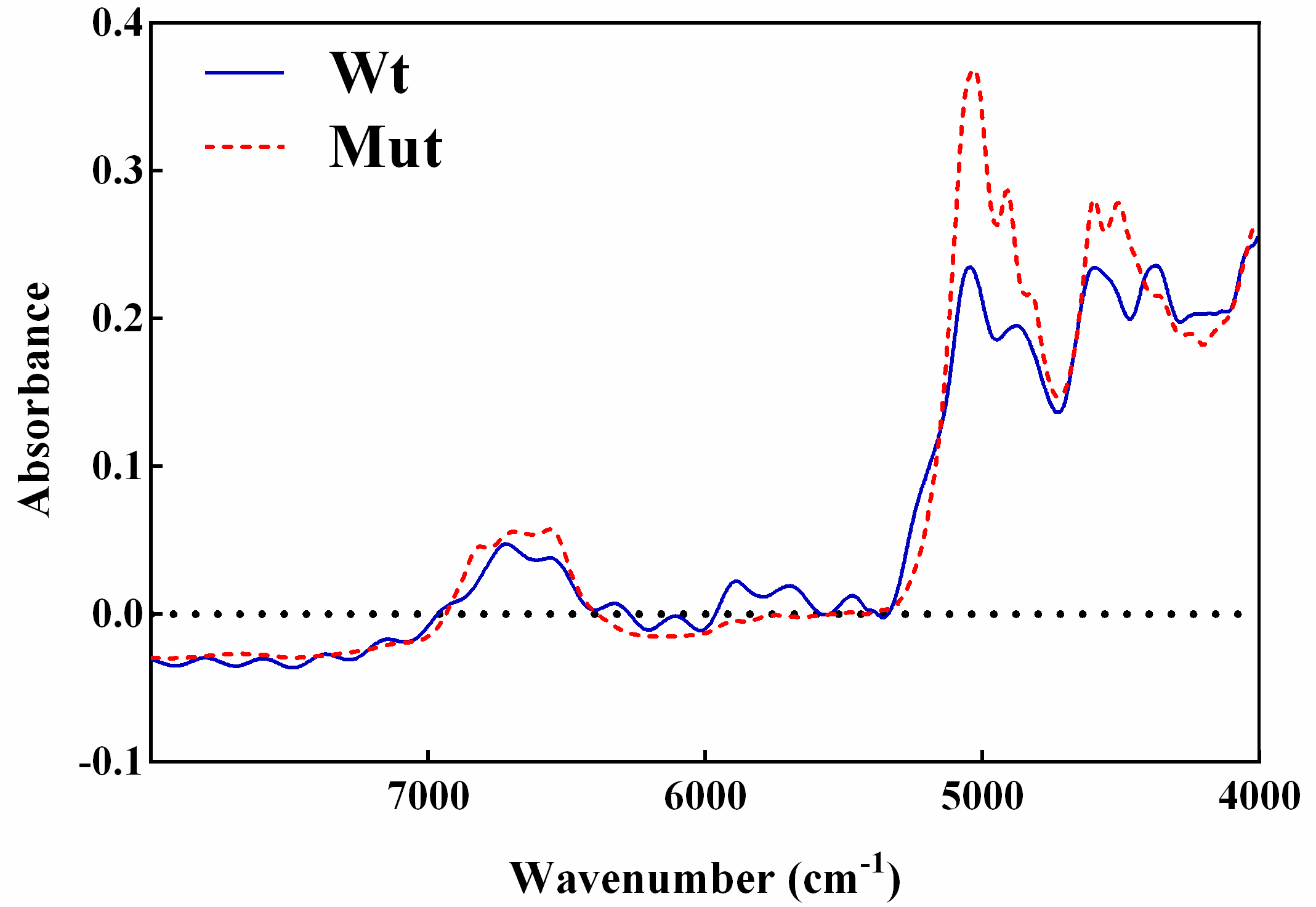

Supplement: S4 Fig — The spectra were recorded at 25 °C in the range of 8,000 to 4,000 cm-1 with a resolution of 8 cm-1. Mut and Wt respectively stand for the mutant and wild-type proteins. (TIF) [file pone.0260306.s004.tif]

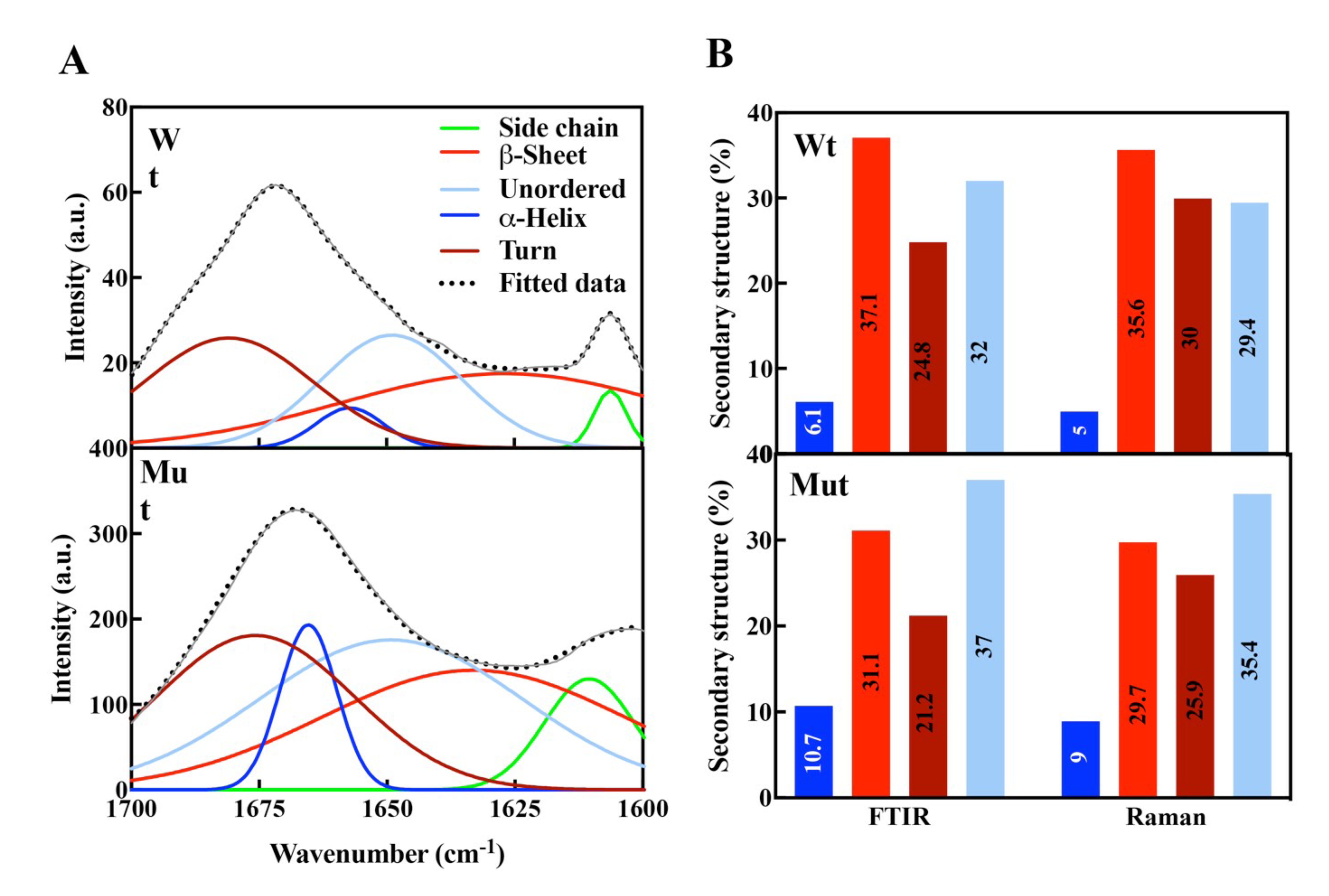

Supplement: S5 Fig — A) Curve fitting and deconvolution analysis of Raman spectra in amide I region were carried out using the peaks as described in FTIR method section. B) Comparison of the secondary structural content of different protein samples by FTIR and Raman spectroscopy. Mut and Wt respectively stand for the mutant and wild-type proteins. (TIF) [file pone.0260306.s005.tif]

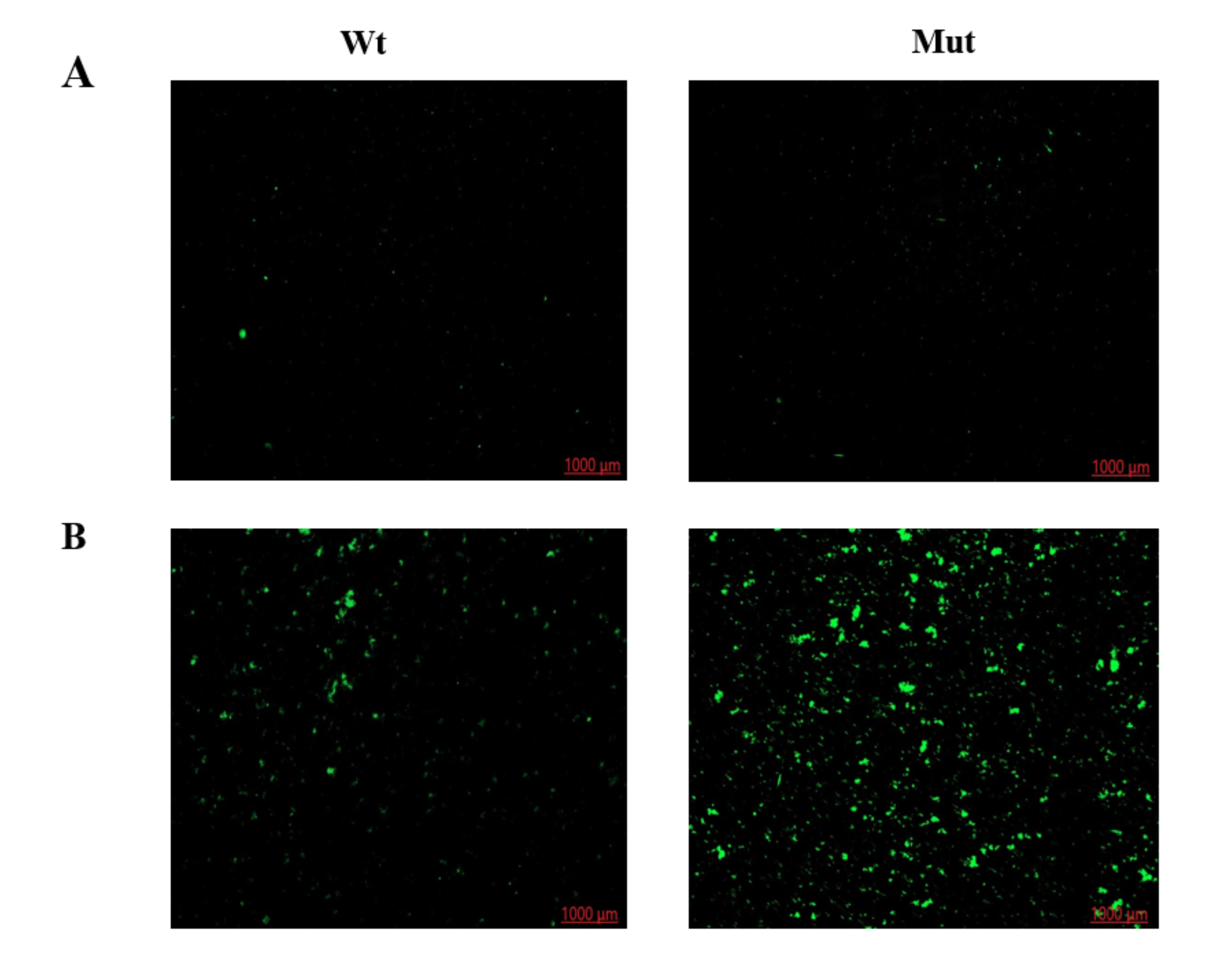

Supplement: S6 Fig — Protein samples (2 mg.mL-1 prepared in buffer A) were incubated at 60 °C for 4 days and after a 5 min incubation of each protein sample (0.15 mg.mL-1) with 20 μM ThT. The fluorescence microscopy studies were used to analyse the fiber plaques of protein samples using the green filter (GFP) with an excitation and emission wavelength of 469 nm and 525 nm, respectively. Mut and Wt respectively stand for the mutant and wild-type proteins. (TIF) [file pone.0260306.s006.tif]

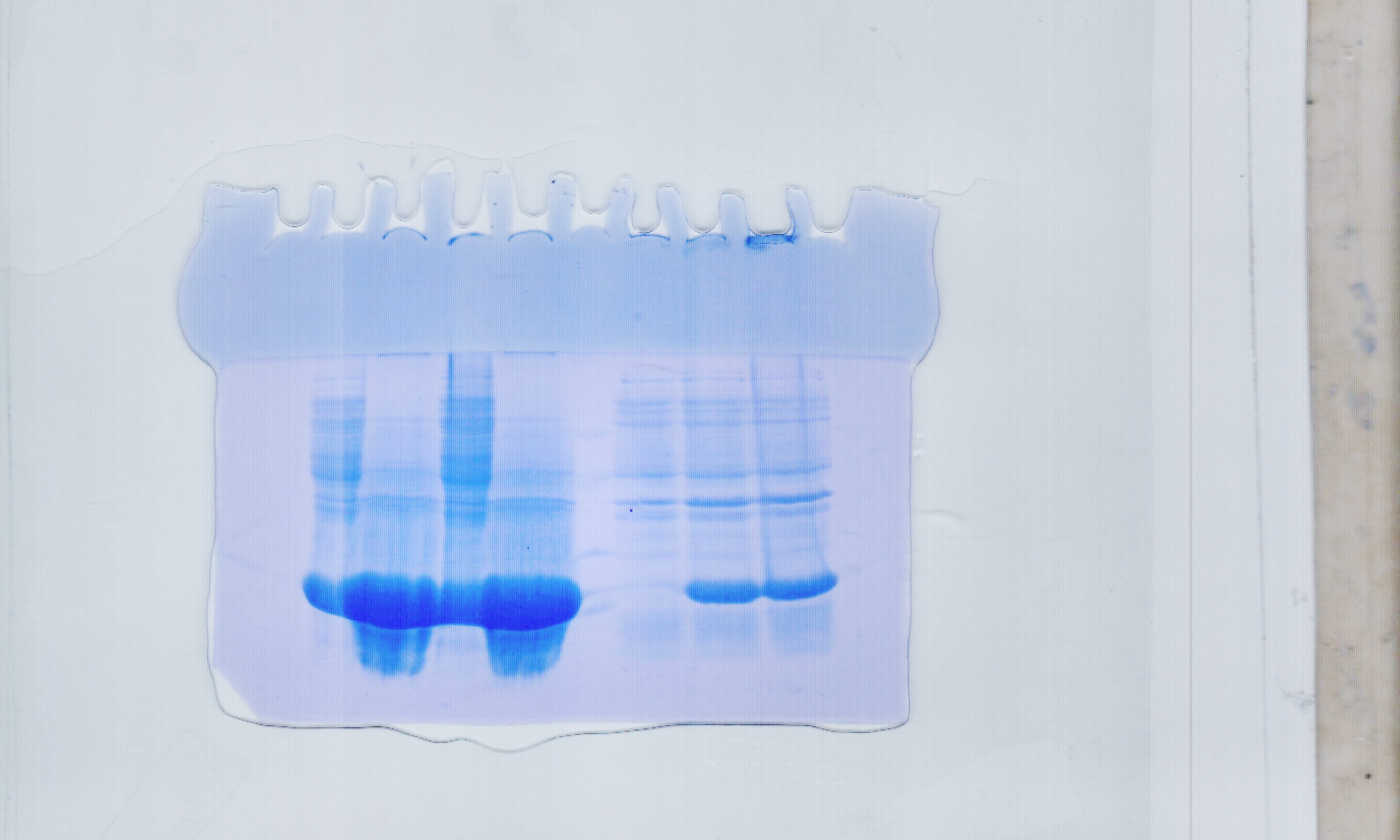

Supplement: S1 Raw images — (ZIP) [file pone.0260306.s007.zip › Fig. 7C_Right.jpg]

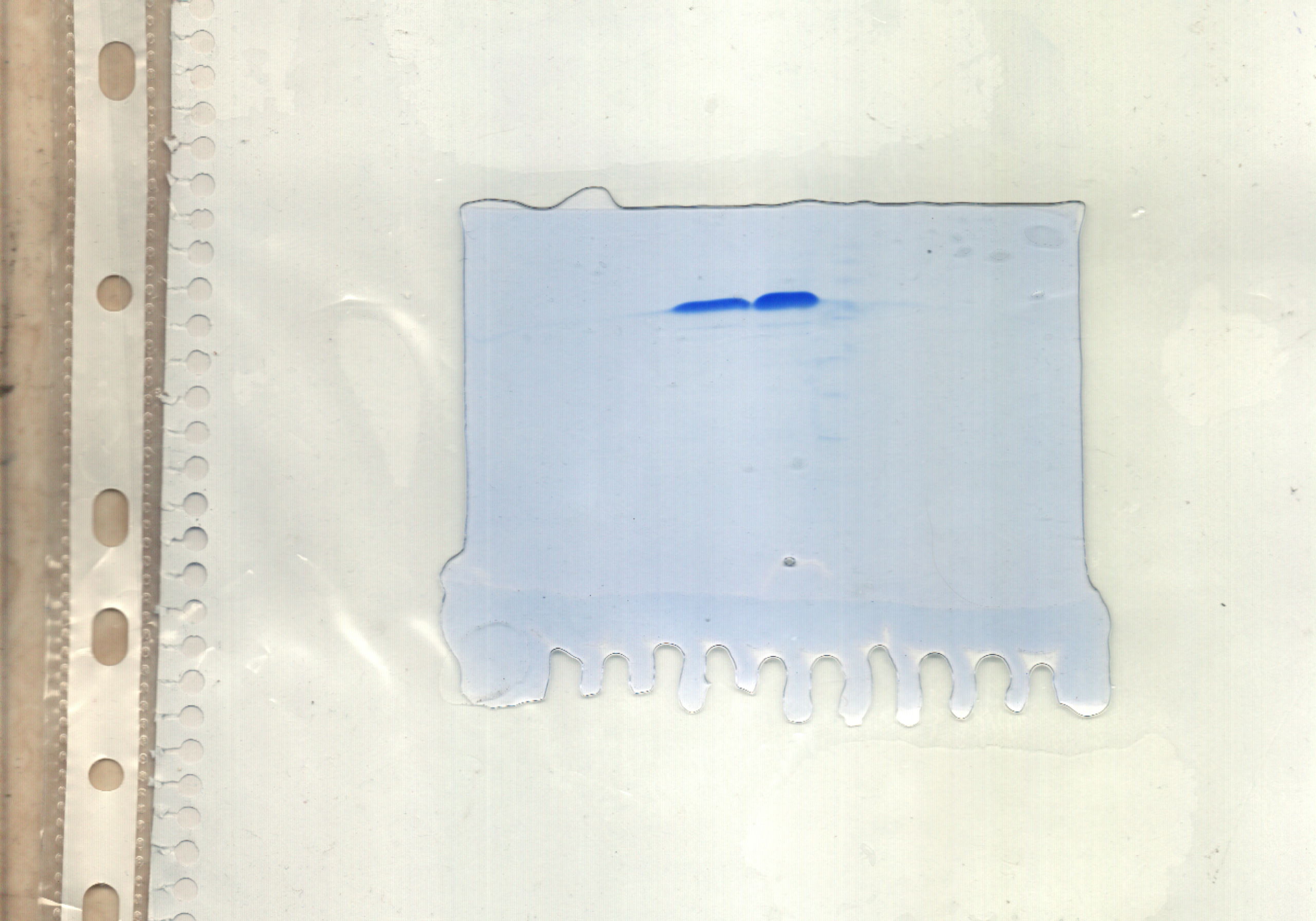

Supplement: S1 Raw images — (ZIP) [file pone.0260306.s007.zip › Fig. S1B.JPG]

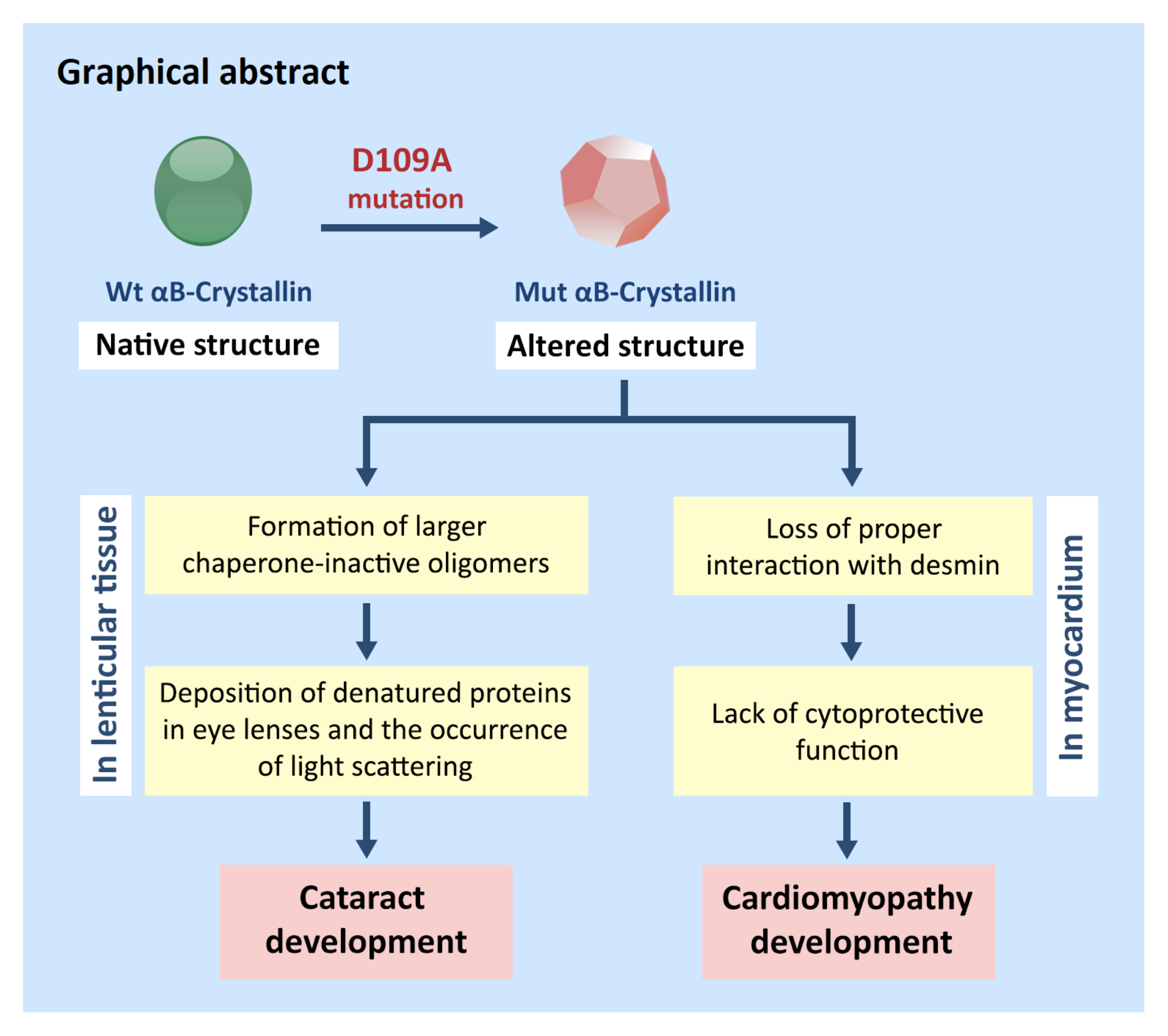

Supplement: S1 Graphical abstract — (TIF) [file pone.0260306.s008.tif]
